# A new DES-mediated synthesis of Henna-based benzopyranophenazines and benzoxanthenetriones

**DOI:** 10.1038/s41598-024-66971-5

**Published:** 2024-07-15

**Authors:** Arezo Monem, Davood Habibi, Hadis Goudarzi

**Affiliations:** Department of Organic Chemistry, Faculty of Chemistry, Bu-Ali Sina University, Hamedan, 6517838683 Iran

**Keywords:** Benzopyranophenazines, Benzo-xanthenetriones, Deep eutectic solvent, Henna-based compounds, MCRs, Chemistry, Materials science

## Abstract

MTPPBr/THFTCA-DES was prepared as a new deep eutectic solvent (DES) from a mixture (molar ratio 7:3) of methyltriphenyl-phosphonium bromide (MTPPBr) and tetrahydrofuran-2,3,4,5-tetra-carboxylic acid (THFTCA), and characterized with various spectroscopic techniques, densitometer, and eutectic point. Then, it was used as a new and powerful catalyst for the synthesis of two sets of biologically important compounds, namely the Henna-based benzopyranophenazines and benzoxan-thenetriones. Solvent-free conditions, short reaction time, high efficiency, and easy recycling and separation of the DES catalyst are among the most important features of the presented method. Also, there is a nice consistency between the proposed structure of the DES compound, the integration values of the ^1^H NMR peaks, and the ratio of MTPPBr to THFTCA obtained from the eutectic point phase diagram. In addition, the reduction of peak splitting patterns in DES compared to the two primary materials can be good evidence of the formation of hydrogen bonds between the two components.

## Introduction

The importance of preserving the environment is followed seriously and green chemistry has taken a smooth path to achieve these goals. The use of targeted solvents and catalysts is a fundamental solution in pursuing environmental goals^1,2^. Ionic liquids have proven their importance for these purposes and have attracted the attention of many research groups^3,4^. DESs have been developed in line with the goals of green chemistry as a suitable alternative to ionic liquids (ILs) and have found many applications in various research fields^5^. DES is a mixture of two or more ionic compounds with a lower melting point than the starting materials (often cheap and safe) and was first reported in 2003 by the Abbott Research Group^6–11^. In recent years, DESs have become a very suitable substitute for other organic solvents due to their simple preparation via using available compounds^12,13^. These compounds are usually used in organic reactions and can act as both a solvent and a catalyst. The catalytic activity of these new ionic liquids is due to their hydrogen bonding properties^14^. As mentioned, these compounds usually consist of a mixture of at least two components. In the structure of these compounds, there is a hydrogen bond acceptor (HBA) and a hydrogen bond donor (HBD), which can create a new eutectic phase with a melting point lower than the melting point of each component^15^. These compounds have important features such as high safety, suitable renewable, low toxicity, biodegradability, and low cost, which increases the importance of designing and synthesizing these compounds^16,17^.

Many DESs are safe and environmentally friendly liquids whose ability to give or accept electrons or protons is their main feature. For this reason, they can be used to dissolve a wide range of compounds such as salts, proteins, drugs, amino acids, surfactants, sugars, and polysaccharides^18^.

Solubility is a crucial factor in drug science. Water is considered a biological solvent, and drug solubility in it is an essential physical and chemical characteristic that determines the design of oral or injectable drug formulations. However, some drugs have little solubility or are insoluble in water^19,20^. Therefore, DESs are used as co-solvents in drug science to increase solubility. For example, Morrison and coworkers reported on the solubility of benzoic acid, danazol, griseofulvin and itraconazole in urea-choline chloride and malonic acid-choline chloride DES. The solubility of these molecules increases, in some cases, 5–22,000-fold when compared with their solubility in water^18,21^. In addition, the presence of DESs significantly increased the solubility of itraconazole, lidocaine, and piroxicam by 6700, 28, and 430 times respectively, compared to water^22^. The study also investigated the solubility of naproxen drug in DESs of choline chloride:urea, choline chloride:ethylene glycol, and choline chloride:malonic acid. The DES containing malonic acid resulted in the highest amount of naproxen dissolution^23^.

Also, DESs dissolve in various metal oxides and can be used in electrochemical processes such as metal recycling, metal finishing (plating or electropolishing), and metal separation^24^.

In addition, in some research, their high ability to dissolve carbon dioxide has been pointed out, and there are reports of it in gas polishing systems, catalysts, and stabilization of chemical compounds from carbon dioxide. DESs are used with the aim of creating an environment to improve the contact between reactants and finally, to expand the production of organic compounds and catalysis (homogeneous or heterogeneous). In addition, they can be used as catalysts as well as in environmentally safe environment in electrophilic substitution, nucleophilic reactions, Diels–Alder condensation, copolymerization, dehydration of carbohydrates, carbon–carbon bond fomation (Hack or Suzuki), and reduction and multicomponent reactions^25–30^.

Multicomponent reactions (MCRs) are reactions in which more than two reactants are combined to produce a product with a very high selectivity in which most atoms of the participating substances are preserved. The advantages of MCRs include mild conditions, short reaction time, environmental friendliness, easy purification of products, and reduction of reactive substances, solvents, and cost, so they are considered valuable tools in total synthesis and drug discovery^31–39^.

In general, heterocyclic moieties exist in the structure of many pharmaceutical and biological compounds that can be designed and synthesized via MCR, of which 1,4-naphthoquinone is a good example^40–42^.

Naphthoquinones have been shown to be antibacterial, antiproliferative, mollusci-cide, anti-leishmania, anti-fungal, anti-malarial, and anti-viral^43–48^. Lawsone (the primary coloring agent found in the henna plant Lawsonia inermis L) is one of the 1,4-naphthoquinones that show significant biological activity. It is used as an anti-fungal, anti-tumor, anti-bacterial, and as a sunscreen in ultraviolet (UV) filters and hair dyes^49–53^. Henna-based compounds such as benzo[*a*]pyrano[2,3-*c*]phenazines, and benzo[*b*]xanthene-triones due to their broad biological properties, such as anti-cancer, spasmolytic, diuretic, anti-coagulant and anti-anaphylactic activity are of significant importance^54–57^. Other biological cores whose biological properties have received much attention are phenazines, xanthenes, and benzo-xanthenes, which have very important biological properties such as pesticides, antiparasitic, antiplatelet, antitumor, anti-malarial, and anti-tuberculosis.

In recent years, various methods have been proposed for synthesizing benzopyranophenazines, including Ni-Gly-Isatin@boehmite^58^, *β*-cyclodextrin^59^, InBr_3_^60^, FeAl_2_O_4_ NP^61^, caffeine^62^, theophylline^63^, GO-HPG-SO_3_H^64^, La@guanine@SBA-15^65^, Ce/PDA/CPTS@CoFe_2_O_4_^67^, and DABCO^68^. For the synthesis of benzoxanthenetriones, methods such as Fe_3_O_4_@SiO_2_/PetOx^69^, H_2_SO_4_/H_2_O^70^, bmim[HSO_4_]^71^, L-proline^72^, PS/GaCl_3_^73^, PTSA^74^, Fe_3_O_4_/PEG/SA^75^, [bmim][Br]^76^, NMe_2_-Pyrrol.-HSO_4_^77^, and Bi(OTf)_3_ have been suggested.

**Scheme 1:**
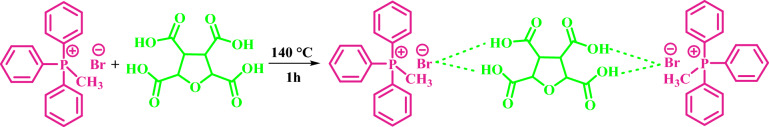
Synthesis of MTPPBr/THFTCA-DES as a new DES.

In continuing our research to find methods for the preparation of the new catalysts, we would like to report the preparation and characterization of a new DES (MTPPBr/THFTCA-DES) from the mixture of MTPPBr and THFTCA (7:3 molar ratio) (Scheme 1).

**Scheme 2:**
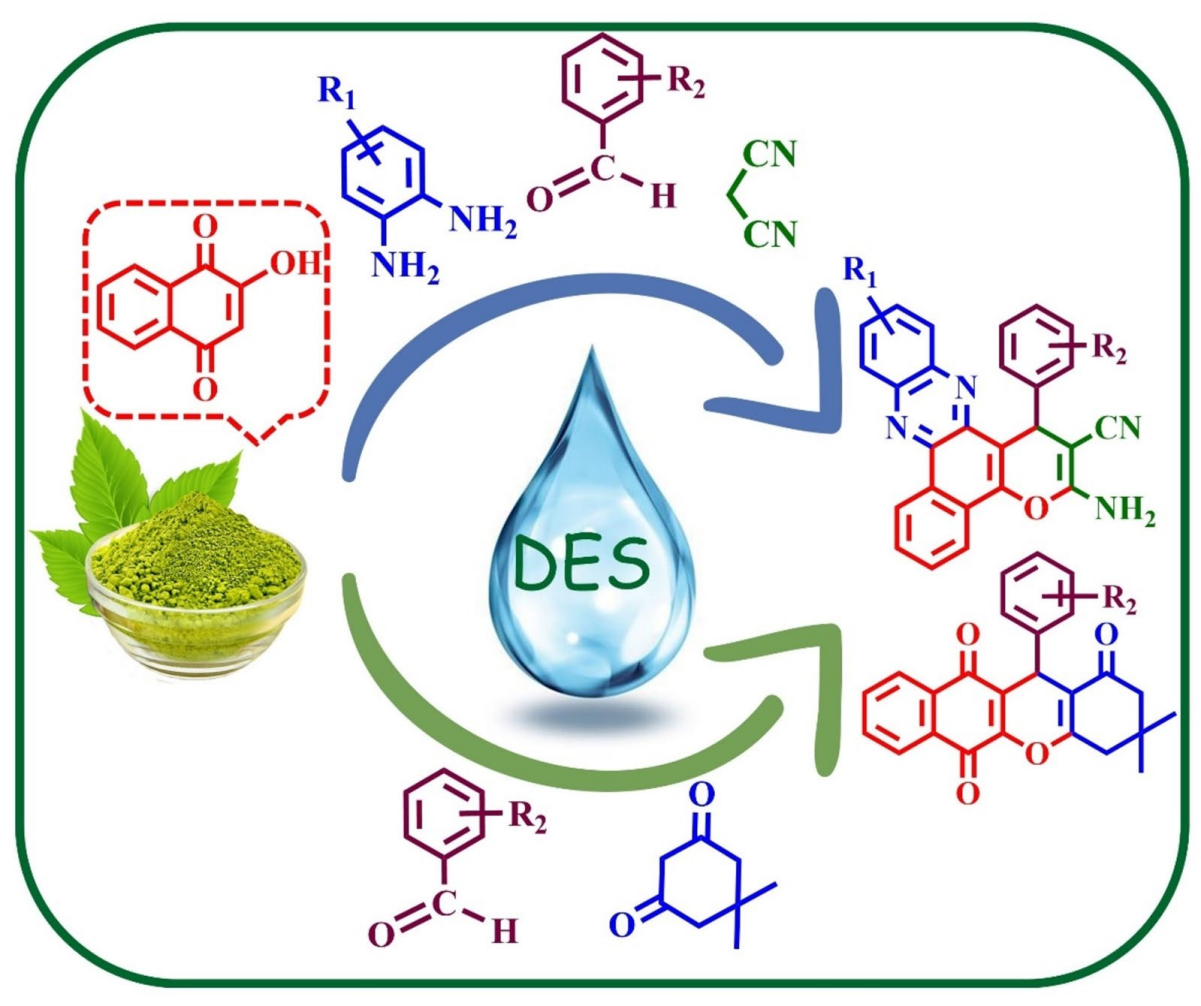
Synthesis of **3(a-v)** and **4(a-l)** by MTPPBr/THFTCA-DES.

Then, the catalytic ability of the new obtained DES was studied in the synthesis of the two sets of the following biological active compounds:

**A)** the ten new **[3(a-j)]** and the twelve known **[3(k-v)]** Henna-based benzopyranophenazines from the reaction of 2‐hydroxynaphthalene‐1,4‐dione, 1,2‐phenylenediamine, aldehyde and malononitrile, and **B)** the twelve known **[4(a-l)]** benzoxanthenes-triones from the reaction of 2‐hydroxynaphtha-lene‐1,4‐dione, aldehyde, and dimedone in a cheap, simple, and non-toxic method with high yields and short reaction times in solvent-free conditions (Scheme 2).

## Experimental

### Materials and methods

Comprehensive information about the starting materials suppliers, and the used scientific equipments for characterization of the DES and all prepared compounds are found in Supporting Information.

#### General procedure for preparation of a new DES compound

The mixture (molar ratio 7:3) of MTPPBr (7 mmol) and THFTCA (3 mmol) were stirred at 140 °C until a homogeneous transparent liquid obtained. Then, H_2_O (20 mL) and EtOAc (20 mL) were added and DES extracted from the aqueous layer, dried at room temperature and stored for further reactions^17^.

#### General procedure for the synthesis of 3(a-v) by DES

DES (1.5 mmol) was added to a mixture of 2-hydroxy-1,4-naphthoquinone (1 mmol), 1,2‐phenylene-diamines (1 mmol), malononitrile (66 mg, 1 mmol), and aldehydes (1.0 mmol), and stirred under solvent‐free conditions at 80 °C for an appropriate time.

After completion of the reaction (TLC, n-hexane/ethyl acetate 8:2), the mixture was washed with ethanol and filtered to separate the catalyst (the reaction mixture is insoluble in ethanol and the DES catalyst is soluble). Ethanol was removed from the filtrate and the DES catalyst stored for further reactions.

A solid precipitate was washed with ethanol and characterized with comparison of their FT-IR, ^1^ H-NMR, ^13^C-NMR, Mass spectra, and melting points with authentic samples.

#### General procedure for the synthesis of 4(a-l) by DES

DES (1.5 mmol) was added to a mixture of 2-hydroxy-1,4-naphthoquinones (1 mmol), dimedone (140 mg, 1 mmol), and aldehydes (1.0 mmol) and stirred under solvent‐free conditions at 80 °C for an appropriate time.

After completion of the reaction (TLC, n-hexane/ethyl acetate 8:2), the mixture was diluted with water (20 mL) and ethyl acetate (20 mL) and shaked (the reaction mixture is insoluble in water and the DES catalyst is soluble). The aqueous layer was separated by, water removed and DES stored for further reactions.

A solid precipitate was washed with water and characterized with a comparison of their FT-IR, ^1^H- NMR, and melting points with authentic samples.

### Ethical approval

Hereby, as a corresponding author, I (Davood Habibi) am responsible for ensuring that all the descriptions in this manuscript are accurate and agreed upon by all authors in such a manner as to meet the standard of the Journal (Research on Chemical Intermediates.

## Results and discussion

DESs, which are an important class of organic compounds, have expanded greatly in recent years as environmentally friendly compounds and acts as both a solvent and a catalyst. The catalytic performance of these compounds is based on the ability to create hydrogen bonds between itself and starting materials.

### Characterization of DES

The new DES catalyst was characterized by different techniques such the FT-IR, eutectic point, ^1^H NMR, TGA/DTA, and densitometer.

### Characterization by FT-IR

**Figure 1:**
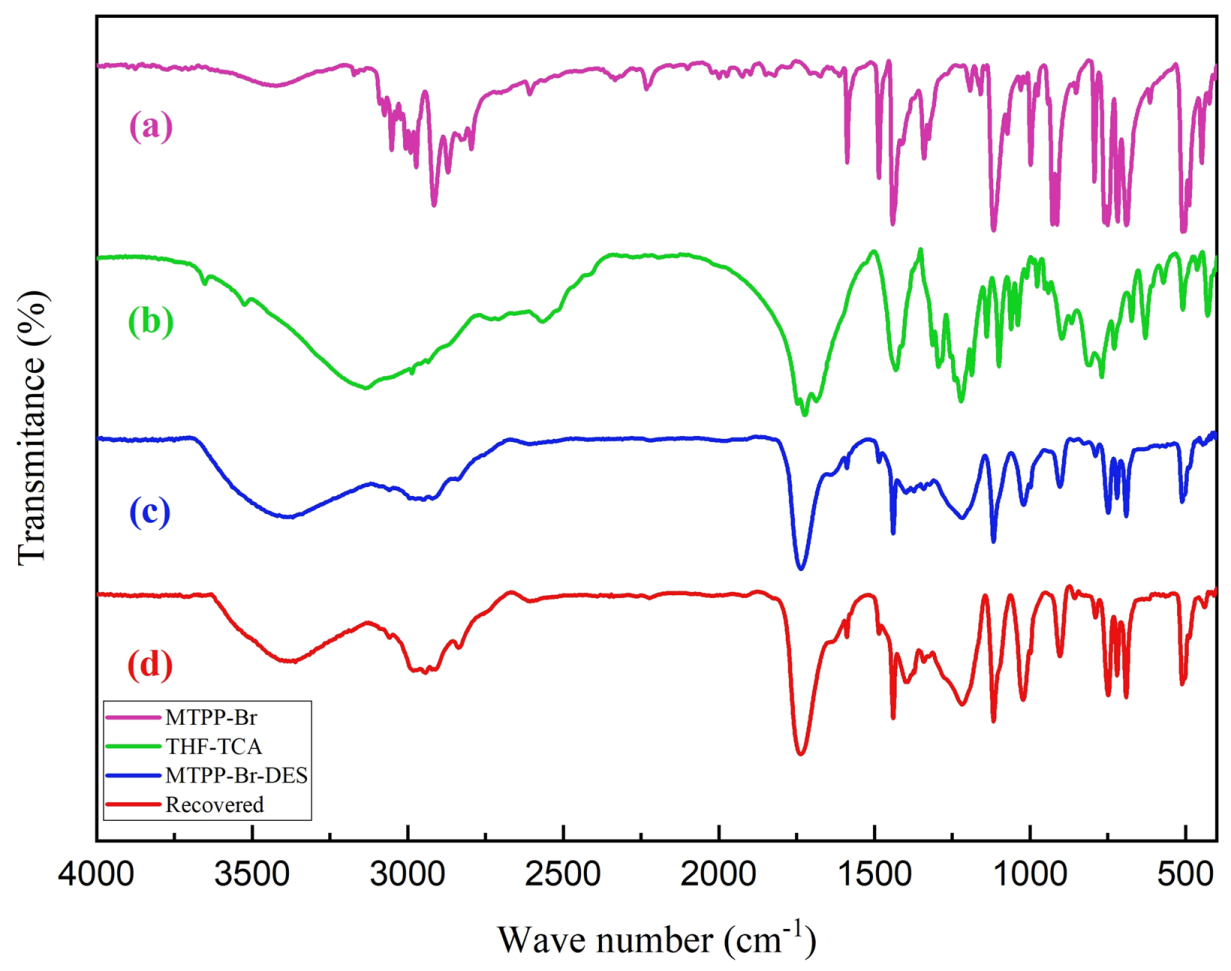
The FT-IR spectra of (**a**–**d**).

Figure 1 shows the FT-IR spectra of MTPPBr (a), THFTCA (b), MTPPBr/THFTCA-DES (c), and the recovered DES catalyst (d). In spectrum (a), the peaks at about 2900–3100 cm^−1^ are related to the aromatic and aliphatic hydrogens, and the peaks at about 750 and 1480 cm^−1^ are related to the C-P bonds, respectively. In spectrum (b), the peaks at 3600 and 1735 cm^−1^ are related to the O–H and C = O of the -COOH group, respectively. In spectrum (c), the above-mentioned peaks can be seen in both (a) and (b) spectra, which confirm the structure of the DES catalyst.

Also, similarity of the IR spectrum of the used DES with the fresh one, confirms the structure of the recovered DES.

### Characterization by eutectic point

**Figure 2:**
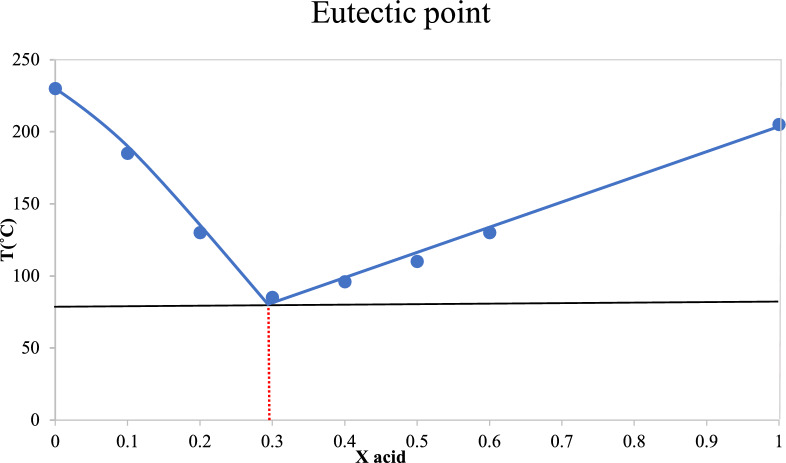
The eutectic points phase diagram of MTPPBr/THFTCA-DES.

To check the best ratio and the chemical composition of the MTPPBr to THFTCA, the eutectic point experiment was performed. So, ten different portions (100–0%) of MTPPBr with a melting point about 230 °C were added to the ten different portions (0–100%) of THFTCA with a melting point about 205 °C and the obtained melting points were recorded each time. Figure 2 shows that the best-obtained ratio of MTPPBr to THFTCA is about 7 to 3 (almost 2:1) with the melting point about 90 °C.

### Characterization by ^1^H NMR

**Figure 3:**
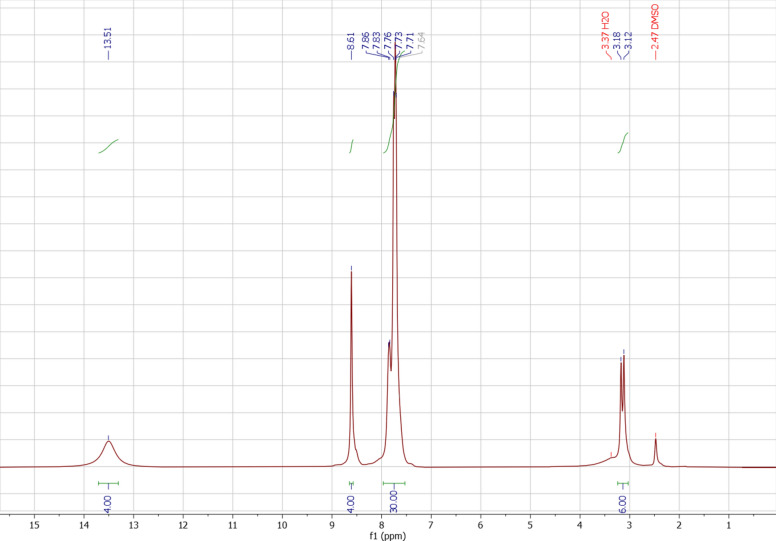
The ^1^H NMR of MTPPBr/THFTCA-DES.

Figure 3 shows the ^1^H NMR spectrum of MTPPBr/THFTCA-DES. The peaks at 3.12–3.18 (d, 6H), 7.85 (m, 30H), 8.61 (s, 4H), and 13.51 ppm (s, 4H) are related to the CH_3_ hydrogens, the phenyl rings, the CH of THFTCA, and the CH of acid (THFTCA), respectively. The decrease in the splitting patterns of the peaks in DES, compared to the raw materials can be very nice evidence of hydrogen bond formation between the two components. The formed hydrogen bonds have caused a gap between the phenyl groups in THFTCA and the methyl groups in MTPPBr, so the long-range coupling is no longer possible.

Also, there is a very nice consistency between the proposed structure for the DES compound (Scheme 1) and the integration values of the ^1^H NMR peaks (Fig. 3). The ratio of the peak integration values of the Hs of the CH_3_ groups, the Hs of the phenyl groups, the acidic Hs of the THF tetra-acid, and the Hs of the THF cycle is 6:30:4:4 indicating the presence of the six hydrogens of the two CH_3_ groups, the thirty hydrogens of the six phenyl groups, the four acidic hydrogens and the four hydrogens of the one THF cycle.

Incidentally, the results obtained from the FT-IR spectrum (Fig. 1), the eutectic points curve (Fig. 2), and the ^1^H NMR integration values (Fig. 3) are almost in the same direction (the ratio of MTPPBr to THFTCA is almost two to one), confirming the proposed structure for the DES compound (Scheme 1).

### Characterization by TGA-DTA

**Figure 4:**
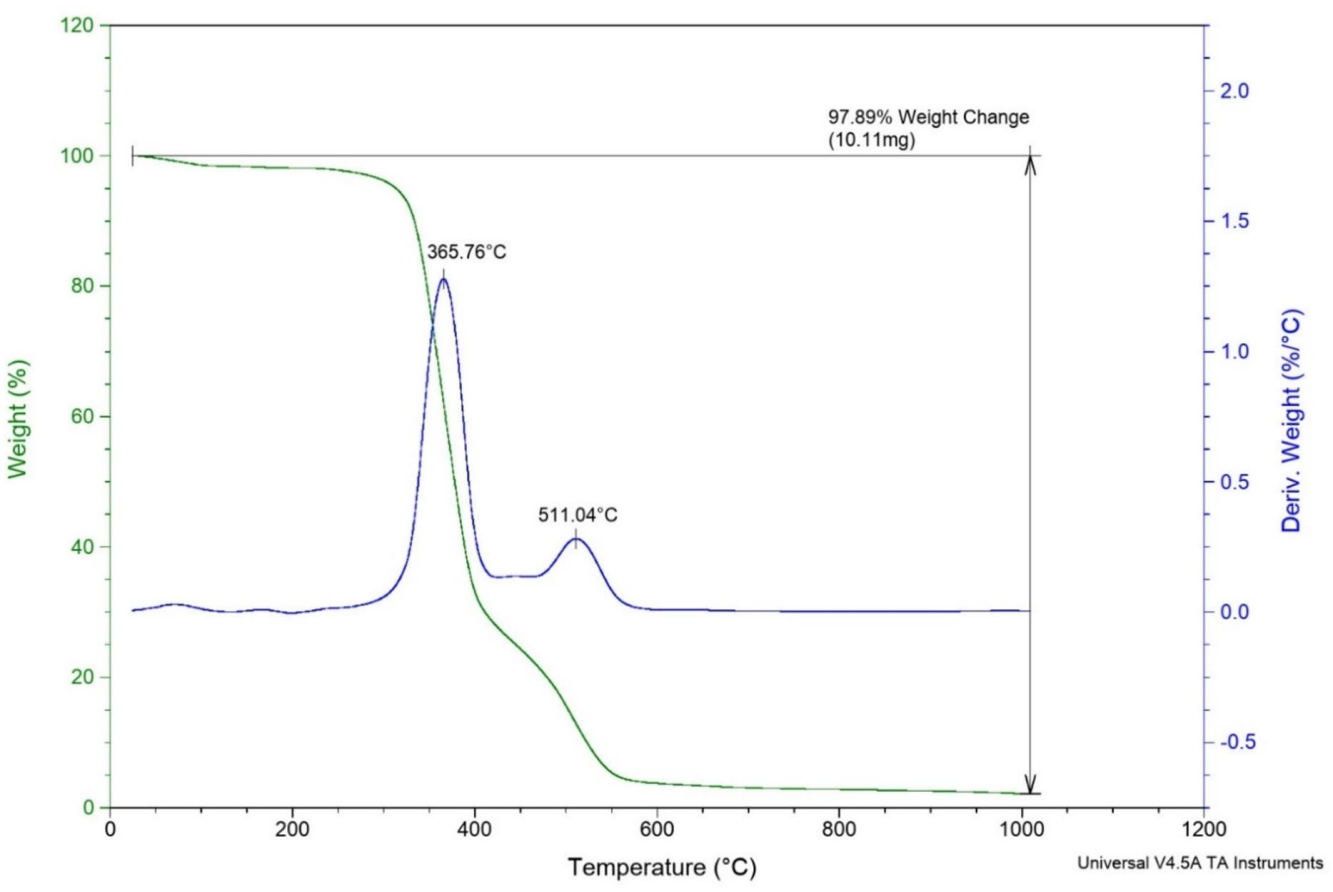
The TGA-DTA patterns of MTPPBr/THFTCA-DES.

Figure 4 shows two weight losses in the 365 and 511 °C regions. The first one is probably related to the breakdown of the hydrogen bonds of acidic compound, and the second one is related to the breakdown of the ionic bonds.

Also, the DTG curve shows two small breaks below 200 °C, which can be related to the removal of small amount of water and organic solvent.

### Characterization by densitometer

Since most DESs are denser than water, a certain amount of DES mixed with a certain volume of water, then by using the formula (P = P_w_/1−m_w_/m_d_), the density of the new prepared DES was calculated (P = P_w_/1−m_w_/m_d_ = 0.99618/1–0.0472/0.1811 = 1.34733 g/mL), which is about 1.34733 g/mL.

### Optimization of the reaction conditions for the synthesis of 3d

The effect of different parameters on the reaction rate was investigated by the use of the model reaction conditions (reaction of 2‐hydroxynaphthalene‐1,4‐dione, 1,2‐phenylenediamine, malononitrile, and benz-aldehyde). So, reactions were performed in various solvents and solvent-free conditions by different amounts of DES at different temperatures.

The best result was found to be the 1:1:1:1 mol ratio of 2‐hydroxynaphthalene‐1,4‐dione, 1,2‐phenyl-enediamine, malono-nitrile, and benzaldehyde with 1.5 mmol of the DES catalyst at 80 °C in solvent-free conditions (Table 1).

**Table 1:** Optimization of the reaction conditions in the synthesis of **3(a-v)** compounds.

Entry	Catalyst (mmol)	Temp. (°C)	Solvent	Yield (%)
1	0.5	80	–	85
2	1.0	"	–	82
3	2.0	"	–	91
4	2.5	"	–	85
5	3.0	"	–	88
6	1.5	60	–	78
7	1"	70	–	81
8	"	90	–	85
9	"	100	–	78
10	"	110	–	70
11	"	Reflux	EtOH	87
12	"	"	H_2_O	82
13	"	"	H_2_O/EtOH	85
14	"	"	EtOAc	80
15	"	"	*n*-Hexane	88
**16**	**"**	**80**	–	**93**

### Synthesis of diverse 3(a-v) compounds (Supp Info)

Based on the results obtained from the optimal conditions (synthesis of **3d**), the condensation reactions of 2-hydroxynaphthalene-1,4-dione, 1,2-phenylenediamines, malononitrile, and aldehydes were carried out for the synthesis of **3(a-v)** in short reaction times and good yields to show the capability of the DES catalyst (Table 2).

**Table 2:** Synthesis of **3(a-v)** by MTPPBr/THFTCA-DES.


Entry	Aldehyde	1,2-Diamine	Product	Time (min.)	Yield (%)	M.P. (°C) Found, literature	TON	TOF
1		** 1a**	** 3a**	35	85	293–295, NEW	56.60	1.61
2		** 1a**	** 3b**	30	92	281–284, NEW	61.33	2.04
3		** 1a**	** 3c**	20	95	311–313, NEW	59.33	3.16
4		** 1a**	** 3d**	20	89	301–303, NEW	59.33	2.96
5		** 1a**	** 3e**	30	94	299–301, NEW	62.66	2.08
6		** 1a**	** 3f**	25	90	284–288, NEW	60.00	2.40
7		** 1a**	** 3g**	30	86	308–310, NEW	57.33	1.91
8		** 1a**	** 3h**	30	92	283–286, NEW	61.33	2.04
9		** 1a**	** 3i**	25	95	271–274, NEW	63.33	2.53
10		** 1a**	** 3j**	25	88	295–298, NEW	58.66	2.34
11		** 1b**	** 3k**	20	85	281–284, 277–280^58^	56.60	2.83
12		** 1b**	** 3l**	15	95	272–276, 268–270^59^	63.30	4.22
13		** 1b**	** 3m**	15	90	298–303, 305–308^59^	60.00	4.00
14		** 1b**	** 3n**	15	93	300–303, 295–298^58^	62.00	4.13
15		** 1b**	** 3o**	20	95	285–287, 285–288^58^	63.30	3.16
16		** 1b**	** 3p**	20	96	267–272, 266–268^59^	64.00	3.20
17		** 1b**	** 3q**	30	89	295–298, 300–302^59^	59.33	1.97
18		** 1b**	** 3r**	20	77	250–253, 249–252^58^	51.30	2.56
19		** 1b**	** 3s**	20	91	284–288, 281–287^58^	60.60	3.03
20		**1b**	** 3t**	30	90	283–285, 280–282^59^	60.00	2.00
21		**1b**	** 3u**	20	87	267–271, 274–277^58^	58.00	2.90
22		** 1b**	** 3v**	30	92	291–294, 293–296^58^	61.30	2.04

### A proposed mechanism for the synthesis of 3(a-v) compounds

**Scheme 3:**
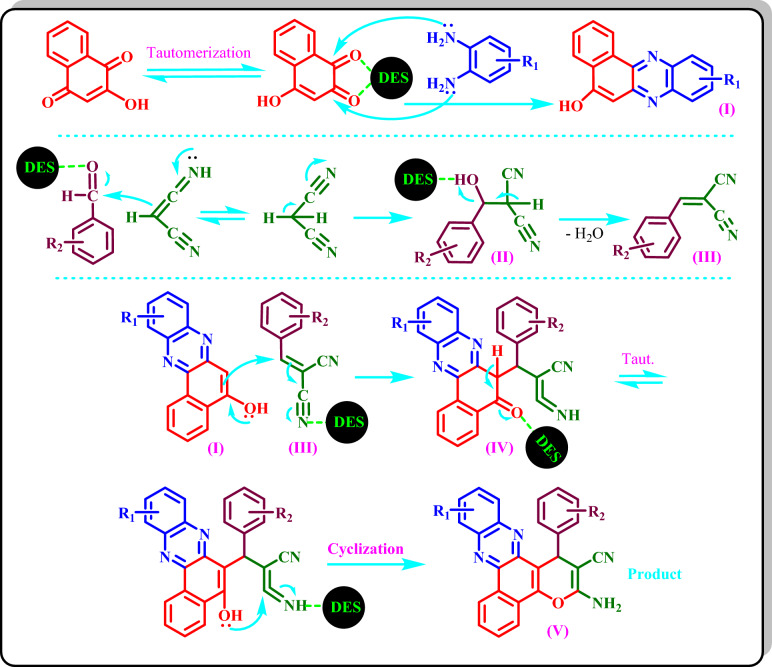
Proposed mechanism for the synthesis of **3(a-v)** by the DES catalyst.

The possible mechanism for the synthesis of **3(a-v)** compounds is shown in Scheme 3. First, the intermediate (I) is formed from the nucleophilic attacks of the two -NH_2_ groups of benzene-1,2-diamine to the two DES activated carbonyl groups of 2-hydroxynaphthalene-1,4-dione. Also, intermediate (III) is formed from the nucleophilic attack of the imine form of malononitrile to the DES activated carbonyl group of an aldehyde with the subsequent deletion of water from intermediate (II). Then, a possible intermediate (IV) is formed via the addition of (I) to (III). Finally, tautomerization of IV and its successive internal cyclization will produce the final product (V).

### Reusability of DES

**Figure 5:**
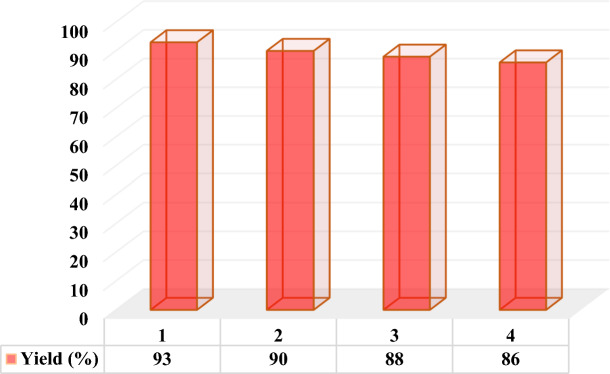
Reusability of the MTPPBr/THFTCA-DES catalyst in four consecutive reaction runs.

After completion of the model reaction (compound **3n**) at the optimum point, the reaction mixture was diluted with water and chloroform and shaked. Then, the aqueous layer containing the DES catalyst was separated from the organic layer by simple liquid–liquid extraction, dried, and reused in consecutive runs without any significant loss of the catalytic activity (95, 90, 88, and 86%, respectively), confirming the stability of the DES catalyst (Fig. 5).

### Comparison of the catalyst activities

Table 3 shows the higher efficiency and better performance of the DES catalyst, compared with the other reported catalysts in the synthesis of **3(a-v)** compounds.

**Table 3:** Comparison of the new DES catalyst with the other reported catalysts in the synthesis of **3(a-v)**.

Entry	Catalyst	Condition	Time (min.)	Yield (%)	Ref
1	Ni-Gly-Isatin@boehmite	PEG, 100 °C	45	96	^58^
2	InBr_3_	EtOH, reflux	50	96	^60^
3	FeAl_2_O_4_ NP	PEG, 100 °C	240	85	^61^
4	Caffeine	EtOH, reflux	50	90	^62^
5	Theophylline	Solvent-free, 70 °C	45	90	^63^
6	GO-HPG-SO_3_H	Solvent-free, 100 °C	45	91	^64^
7	β-cyclodextrin	EtOH /Water, 70 °C	60	89	^59^
8	La@guanine@SBA-15	EtOH, Reflux	180	98	^65^
9	Ce/PDA/CPTS@CoFe_2_O_4_	EtOH/H_2_O, 80 °C	30	97	^66^
10	DABCO	EtOH, Reflux	300	91	^67^
11	This work	Solvent-free, 80 °C	15	93	–

### Optimization of the reaction conditions for the synthesis of 4f

The effect of different parameters on the reaction rate was investigated by the use of the model reaction conditions (reaction of 2‐hydroxynaphthalene‐1,4‐dione, dimedone and benzaldehyde). So, reactions were performed in various solvents and solvent-free conditions by different amounts of DES at different temperatures.

The best result was found to be the 1:1:1 mol ratio of 2‐hydroxynaphthalene‐1,4‐dione, dimedone, and benzaldehyde with 1.5 mmol of the DES catalyst at 80 °C in solvent-free conditions with high yield in short reaction time (Table 4).

**Table 4:** Optimization of the reaction conditions for the synthesis of **4(a-l).**

Entry	Catalyst (mmol)	Temp. (°C)	Solvent	Yield (%)
1	0.5	80	–	85
2	1.0	"	–	90
3	2.0	"	–	87
4	2.5	"	–	85
5	3.0	"	–	88
6	1.5	60	–	88
7	"	70	–	90
8	"	90	–	83
9	"	100	–	80
10	"	110	–	76
11	"	Reflux	EtOH	90
12	"	"	H_2_O	45
13	"	"	H_2_O/EtOH	50
14	"	"	EtOAc	65
15	"	"	*n*-Hexane	73
**16**	**"**	**80**	–	**95**

### Synthesis of diverse 4(a-l) compounds (Supp Info)

Based on the results obtained from the optimal conditions (synthesis of **4f**), the condensation reaction 2‐hydroxynaphthalene‐1,4‐dione, dimedone, and various aldehydes were carried out to synthesize a wide range of the **4(a-l)** compounds. The products were synthesized in a short reaction time and good yields to show the catalytic capability of the MTPPBr/THFTCA-DES compound (Table 5).

**Table 5:** Synthesis of **4(a-l)** compounds using the MTPPBr/THFTCA-DES catalyst.


Entry	Aldehyde	Product	Time (min.)	Yield (%)	M.P. (°C) Found, literature^68–70^	TON	TOF
1		** 4a**	15	90	220–225, 224–226	60.00	4.00
2		** 4b**	25	89	223–226, 221–224	59.33	2.37
3		** 4c**	10	94	233–236, 230–232	62.66	6.26
4		** 4d**	15	95	235–237, 234–236	63.33	4.22
5		** 4e**	20	96	260–264, 256–258	64.00	3.20
6		** 4f**	10	95	228–233, 230–232	63.33	6.33
7		** 4g**	15	88	246–249, 242–24	58.66	3.91
8		** 4h**	10	90	240–246, 238–240	60.00	6.00
9		** 4i**	10	95	200–204, 194–196	63.33	6.33
10		** 4j**	15	82	270–274, 268–270	54.66	3.64
11		** 4k**	20	93	173–177, 168–170	62.00	3.10
12		** 4l**	15	85	202–205, 198–200	56.66	3.77

### A proposed mechanism for the synthesis of 4(a-l) compounds

**Scheme 4:**
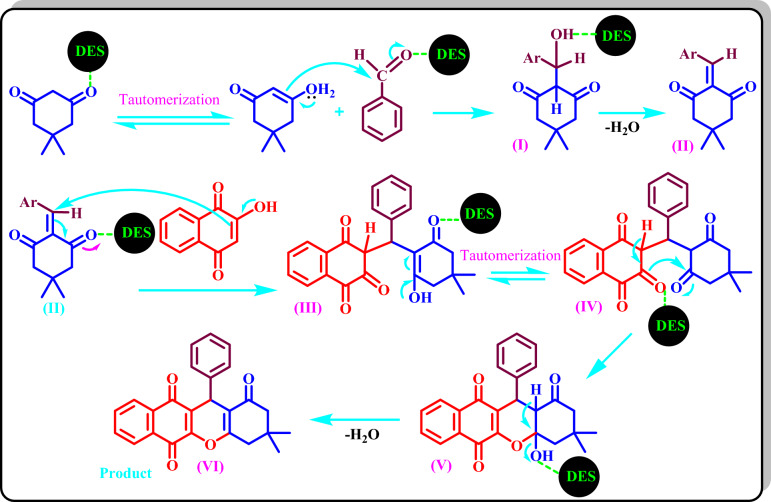
Proposed mechanism for the synthesis of **4(a-l)** by the new DES catalyst.

The possible mechanism for the synthesis of **4(a-l)** is shown in Scheme 4. The DES catalyst activates the carbonyl group of the aldehyde for the nucleophilic attack of dimedone (or 1,3‐dicarbonyl compound) on it to form (I) with the subsequent release of water to form (II). The reaction is continued by the Michael's addition of 2‐hydroxy‐1,4-naphthoquinones to (II) to form (III). The intermediate (IV) is formed by the keto-enol tautomerization of (III), which its intermolecular cyclization will form (V). The final product (VI) is formed by removing water from (V).

### Reusability of DES

**Figure 6:**
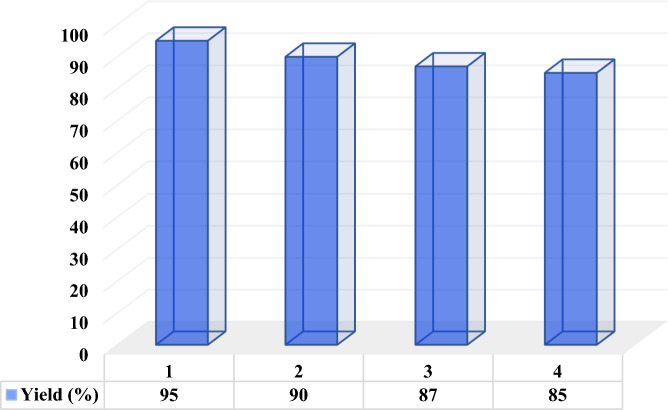
Reusability of the MTPPBr/THFTCA-DES catalyst in four-consecutive reaction runs.

After completion of the model reaction (compound **4f**) at the optimum point, the reaction was stopped and the mixture was diluted with water and chloroform and shaked vigorously. The DES catalyst inside the aqueous layer was separated from the organic layer by simple liquid–liquid extraction and dried to remove water. Then, it was reused in consecutive runs which showed a small decrease in the catalytic activity (90, 87, 85, and 80%, respectively), confirming the stability of the DES catalyst (Fig. 6).

### Comparison of the DES catalyst with other catalysts

Table 6 shows the higher efficiency and better performance of the DES catalyst, compared with the other reported catalysts in the synthesis of **4(a-l)** compounds.

**Table 6:** Comparison of the new DES catalyst with the other reported catalysts in the synthesis of **4(a-l)**.

Entry	Catalyst	Condition	Time (min)	Yield (%)	Ref
1	L-proline	Water, reflux	120	70	^71^
2	PS/GaCl_3_	EtOH, reflux	120	89	^72^
3	PTSA	Water, reflux	840	84	^73^
4	Fe_3_O_4_/PEG/SA	EtOH, ultrasound	20	90	^74^
5	[bmim][Br]	Solvent-free, 90 °C	540	80	^75^
6	bmim[HSO_4_]	Solvent-free, 70 °C	30	91	^70^
7	H_2_SO_4_/H_2_O	100 °C	25	92	^69^
8	NMe_2_-Pyrrol.-HSO_4_	100 °C	25	93	^76^
9	Fe_3_O_4_@SiO_2_/PEtOx	EtOH, reflux	135	82	^68^
10	Bi(OTf)_3_	EtOH, 80 °C	360	89	^77^
11	This work	Solvent-free, 80 °C	10	95	–

## Conclusion

In summary, DESs have recently gained attention as a cost-effective and environmentally friendly alternative to organic synthesis. They can be easily synthesized by mixing and heating the components without the need for purification, and making them energy-efficient. DES not only act as a solvent but also as a recyclable and reusable organocatalyst, therefore, it can play a dual role in organic transformations. The use of DES catalysts has many advantages such as reasonable yields of desired products, short reaction times, low costs of starting materials for catalyst preparation, mild reaction media, and versatile work-up procedures. Using DES with appropriate features, we can develop test protocols that are environmentally friendly, cost effective, and maximally efficient.

In conclusion, a new DES was prepared from a 7:3 mixture of MTPPBr and THFTCA and its structure confirmed by different techniques. The ratio of the peak integration values of Hs of the CH_3_ groups, Hs of the phenyl groups, Hs of the tetra acid, and Hs of the THF cycle is 6:30:4:4 indicating the presence of the two CH_3_ groups, the six phenyl groups, the four acidic hydrogens, and the one THF cycle. Incidentally, the decrease in splitting patterns of the peaks in DES, compared to the two starting materials can be very nice evidence of hydrogen bond formation between the two components.

Then, the new DES compound was used as an efficient, capable, and recyclable catalyst based on its ability to form hydrogen bonds in the synthesis of Henna-based benzopyranophenazines and benzo-xanthenetriones with short reaction times, the high yields. In addition, the new DES compound not only plays the role of the catalyst, but also plays the role of a solvent to provide a mild environmental condition for starting materials to perform the expected reactions.

### Supplementary Information

Supplementary Information.

## Data Availability

All data generated or analyzed during this study are included in this published article and its supplementary information files.
