# Supplementary material for: A new DES-mediated synthesis of Henna-based benzopyranophenazines and benzoxanthenetriones
Source: Sci Rep. 2024 Jul 15;14:16273. doi: 10.1038/s41598-024-66971-5 (PMC11251052; doi:10.1038/s41598-024-66971-5)
Supplement: Supplementary file 1 — Supplementary Information. [file 41598_2024_66971_MOESM1_ESM.docx]

**Supporting Information**

**(Scientific Reports)**

**A new DES-mediated synthesis of Henna-based benzopyranophenazines and benzoxanthenetriones**

**Arezo Monem, Davood Habibi,* Hadis Goudarzi**

Department of Organic Chemistry, Faculty of Chemistry, Bu-Ali Sina University, Hamedan 6517838683, Iran

*Corresponding author email: [davood.habibi@gmail.com](mailto:davood.habibi@gmail.com) (& dhabibi@basu.ac.ir), Tel: +98 81 38380922; Fax: +98 81 31408025

**Content Page**

Materials and methods 3

Spectral data 3

FT-IR spectrum of 3a 15

^1^H NMR spectrum of 3a 15

^13^C NMR spectrum of 3a 16

Mass spectrum of 3a 16

FT-IR spectrum of 3b 17

^1^H NMR spectrum of 3b 17

^13^C NMR spectrum of 3b 18

Mass spectrum of 3b 18

FT-IR spectrum of 3c 19

^1^H NMR spectrum of 3c 19

^13^C NMR spectrum of 3c 20

Mass spectrum of 3c 20

FT-IR spectrum of 3d 21

^1^H NMR spectrum of 3d 21

^13^C NMR spectrum of 3d 22

Mass spectrum of 3d 22

FT-IR spectrum of 3e 23

^1^H NMR spectrum of 3e 23

^13^C NMR spectrum of 3e 24

Mass spectrum of 3e 24

FT-IR spectrum of 3f 25

^1^H NMR spectrum of 3f 25

^13^C NMR spectrum of 3f 26

Mass spectrum of 3f 26

FT-IR spectrum of 3g 27

^1^H NMR spectrum of 3g 27

^13^C NMR spectrum of 3g 28

Mass spectrum of 3g 28

FT-IR spectrum of 3h 29

^1^H NMR spectrum of 3h 29

^13^C NMR spectrum of 3h 30

Mass spectrum of 3h 30

FT-IR spectrum of 3i 31

^1^H NMR spectrum of 3i 31

^13^C NMR spectrum of 3i 32

Mass spectrum of 3i 32

FT-IR spectrum of 3j 33

^1^H NMR spectrum of 3j 33

^13^C NMR spectrum of 3j 34

Mass spectrum of 3j 34

FT-IR spectrum of 3q 35

^1^H NMR spectrum of 3q 35

FT-IR spectrum of 4a 36

^1^H NMR spectrum of 4a 36

FT-IR spectrum of 4b 37

^1^H NMR spectrum of 4b 37

**Experimental**

Materials and methods

The comprehensive information about the starting materials suppliers, and scientific equipments which were used for the characterization can be found in Supporting Information section.

All reagents were purchased from the Merck and Sigma Aldrich chemical companies and used without further purification. Analytical thin-layer chromatography (TLC) was conducted on pre-coated TLC plates; silica gel 60 F-254 (E. Merck, Darmstadt, Germany). The ^1^H NMR (250 MHz, DMSO-*d_6_*) and ^13^C NMR (62.5 MHz, DMSO-*d_6_*) were recorded on a Bruker DRX‐250. FT‐IR (KBr) spectra were recorded on an Alpha Perkin Elmer spectrophotometer. Melting points were taken in open capillary tubes with a Stuart melting point apparatus and are uncorrected. Density analysis was done using the AND-HR200 model solid material density measuring device.

Spectral data of **3(a-v)** compounds

3-Amino-12-chloro-1-(3-nitrophenyl)-1*H*-benzo[*a*]pyrano[2,3-*c*]phenazine-2-carbonitrile (**3a,** new)

Yellow solid, M.P.: 293-295 °C; IR (KBr) ν = 3465, 3342, 3191, 3084, 2200, 1671, 1602, 1523, 1383, 1347, 1268, 1165, 1058, 923, 835, 736 and 705 cm^-1^. ^1^H NMR δ = 9.13 (d, *J* = 7.9 Hz, 1H), 8.43 (d, *J* = 7.8 Hz, 1H), 8.33-8.20 (m, 2H), 8.14 (d, *J* = 12.3 Hz, 1H), 8.09-7.79 (m, 5H), 7.56 (s, 3H), 5.76-5.50 (m, 1H). ^13^C NMR δ = 160.3, 140.6, 135.2, 131.5, 130.4, 128, 125.5, 122.3, 92, 75.8, 57.3, 37.9 and 25.7; MS: m/z = 479 [M]^+^, base peak: m/z = 64.

**3a**

3-Amino-12-chloro-1-(2-methoxyphenyl)-1*H*-benzo[*a*]pyrano[2,3-*c*]phenazine-2-carbo-nitrile (**3b,** new)

Yellow solid, M.P.: 281-284 °C; IR (KBr) ν = 3463, 3344, 3192, 2191, 1671, 1603, 1491, 1469, 1385, 1252, 1060, 1024, 921 and 753 cm^-1^; ^1^H NMR δ = 9.19 (d, *J* = 7.8 Hz, 1H), 8.45 (d, *J* = 7.9 Hz, 1H), 8.30 (s, 1H), 8.07-7.83 (m, 4H), 7.20 (s, 2H), 7.08 (q, *J* = 7.5 Hz, 2H), 6.93 (d, *J* = 7.8 Hz, 1H), 6.74 (d, *J* = 7.2 Hz, 1H), 5.77 (s, 1H), 3.81 (s, 3H); ^13^C NMR δ = 160.6, 157.4, 147.7, 141.8, 140.5, 134.8, 132.3, 131.8, 131.6, 131, 130.1, 129.5, 128.3, 128, 125, 124.5, 122.6, 120.9, 113.9, 112.1, 57.1, 56.3, 40.9, 40.6, 40.3, 39.9, 39.6, 39.3, 38.9 and 32.6; MS: m/z = 464 [M]^+^, base peak: m/z = 64 and 80.

**3b**

3-Amino-12-chloro-1-(2,4-dichlorophenyl)-1*H*-benzo[*a*]pyrano[2,3-*c*]phenazine-2-carbonitrile (**3c,** new)

Yellow solid, M.P.: 311-313 °C; IR (KBr) ν = 3479, 3342, 3179, 3080, 2191, 1661, 1624, 1591, 1471, 1387, 1341, 1166, 1064, 831 and 760 cm^−1^; ^1^H NMR δ = 9.01 (d, *J* = 7.5 Hz, 1H), 8.32 (d, *J* = 7.8 Hz, 1H), 8.09 (d, *J* = 7.8 Hz, 1H), 8.0-7.69 (m, 4H), 7.53 (d, *J* = 4.9 Hz, 1H), 7.39 (s, 2H), 7.12 (s, 2H), 5.65 (d, *J* = 7.8 Hz, 1H); ^13^C NMR δ = 159.8, 147.6, 142.2, 141.6, 140.8, 139, 135.7, 135.3, 133.7, 131.7, 131.3, 129.6, 128.6, 128.2, 127.1, 125.7, 125.4, 122.8, 119.9, 112.4, 56.8, 52.5 and 34.6; MS: m/z = 502 [M]^+^, base peak: m/z = 442.

**3c**

3-Amino-12-chloro-1-phenyl-1*H*-benzo[*a*]pyrano[2,3-*c*]phenazine-2-carbonitrile (**3d,** new)

Yellow solid, M.P.: 301-303°C; IR (KBr) ν = 3456, 3320, 3174, 3061, 2189, 1659, 1622, 1592. 1386, 1340, 1267, 1200, 1163. 1063, 759 and 703 cm^-1^; ^1^H NMR δ = 9.00 (t, *J* = 6.9 Hz, 1H), 8.35 (t, *J* = 6.7 Hz, 1H), 8.09 (d, *J* = 6.0 Hz, 1H), 7.88 (dq, *J* = 32.7, 8.3 Hz, 4H), 7.37 (d, *J* = 6.0 Hz, 4H), 7.33-7.15 (m, 2H), 7.06 (d, *J* = 7.0 Hz, 1H), 5.36 (d, *J* = 5.6 Hz, 1H).^13^C NMR δ = 160.2, 145.6, 140.4, 131.3, 129.6, 128.8, 128.1, 127, 125.7, 122.6, 114.1, 58.4 and 37.8; MS: m/z = 434 [M]^+^, base peak: m/z = 358.

**3d**

3-Amino-1-(3-bromophenyl)-12-chloro-1*H*-benzo[*a*]pyrano[2,3-*c*]phenazine-2-carbonitrile (**3e,** new)

Yellow solid, M.P.: 299-301 °C; IR (KBr) ν = 3469, 3324, 3182, 2195, 1662, 1591, 1385, 1057, 831 and 763 cm^−1^; ^1^H NMR δ = 8.86 (d, *J* = 7.9 Hz, 1H), 8.29 (d, *J* = 8.0 Hz, 1H), 7.94-7.72 (m, 5H), 7.54 (d, *J* = 7.1 Hz, 1H), 7.44 (s, 2H), 7.37 (d, *J* = 7.4 Hz, 1H), 7.30 (d, *J* = 7.9 Hz, 1H), 7.20 (d, *J* = 8.0 Hz, 1H), 5.29 (d, *J* = 5.2 Hz, 1H).; ^13^C NMR δ = 160.3, 148.2, 142, 139.2, 131.4, 130.1, 127.6, 125.4, 122.8, 122, 120.4, 118.5, 113.3, 57.7 and 37.7; MS: m/z = 514 [M]^+^, base peak: m/z = 357.

**3e**

3-Amino-12-chloro-1-(2-chlorophenyl)-1*H*-benzo[*a*]pyrano[2,3-*c*]phenazine-2-carbonitrile (**3f,** new)

Yellow solid, M.P.: 284-288 °C; IR (KBr) ν = 3471, 3433, 3322, 3172, 2192, 1660, 1624, 1592, 1403, 1387, 1165, 1063, 759 and 751 cm^-1^; ^1^H NMR δ = 9.12 (d, *J* = 7.7 Hz, 1H), 8.40 (d, *J* = 7.7 Hz, 1H), 8.19 (d, *J* = 10.2 Hz, 1H), 8.00 (s, 1H), 7.88 (dt, *J* = 13.5, 8.7 Hz, 3H), 7.36 (d, *J* = 8.2 Hz, 3H), 7.26-7.11 (m, 2H), 7.08 (d, *J* = 4.6 Hz, 1H), 5.82 (d, *J* = 6.8 Hz, 1H); ^13^C NMR δ = 159.9, 149.7, 141.5, 135, 131.4, 129.9, 128.1, 126.3, 125.8, 122.8, 113.7, 65.2 and 33.9; MS: m/z = 468 [M]^+^, base peak: m/z = 357.

**3f**

3-Amino-12-chloro-1-(4-nitrophenyl)-1*H*-benzo[*a*]pyrano[2,3-*c*]phenazine-2-carbonitrile (**3g,** new)

Yellow solid, M.P.: 308-310 °C; IR (KBr) ν = 3459, 3353, 3199, 3077, 2194, 1668, 1604,1512, 1470, 1395, 1344, 1167, 1059, 922, 834 and 759 cm^-1^; ^1^H NMR δ = 8.91 (d, *J* = 7.9 Hz, 1H), 8.32 (d, *J* = 8.0 Hz, 1H), 8.07 (dd, *J* = 8.8, 2.7 Hz, 2H), 7.98-7.83 (m, 4H), 7.78-7.70 (m, 1H), 7.61 (dd, *J* = 8.6, 4.4 Hz, 2H), 7.48 (s, 2H), 5.37 (d, *J* = 4.0 Hz, 1H); ^13^C NMR δ = 160, 153.1, 146.6, 140, 135, 131.5, 131, 129.5, 127.5, 127.2, 125.8, 125.1, 124, 122.6, 120.3, 112.2, 57.2 and 38; MS: m/z = 479 [M]^+^, base peak: m/z = 357.

**3g**

3-Amino-12-chloro-1-(3,4-dimethoxyphenyl)-1*H*-benzo[*a*]pyrano[2,3-*c*]phenazine-2-carbonitrile (**3h,** new)

Yellow solid, M.P.: 283-286 °C; IR (KBr) ν = 3416, 3336, 2944, 2834, 2193, 1663, 1606, 1514, 1418, 1385, 1264, 1027 and 758 cm^-1^; ^1^H NMR δ = 9.17 (d, *J* = 7.9 Hz, 1H), 8.43 (d, *J* = 7.8 Hz, 1H), 8.32-8.14 (m, 2H), 7.94 (q, *J* = 9.0 Hz, 3H), 7.34 (s, 2H), 7.16 (d, *J* = 10.2 Hz, 1H), 6.80 (dd, *J* = 7.5, 5.1 Hz, 2H), 5.45 (d, *J* = 3.1 Hz, 1H), 3.70 (s, 3H), 3.59 (s, 3H).; ^13^C NMR δ = 160.4, 148.6, 147.9, 146.8, 140.5, 138, 136.2, 135, 132, 131.4, 130.9, 129.6, 128, 125.5, 122.7, 120.7, 119.9, 114.6, 112.3, 58.3, 55.8 and 37.2; MS: m/z = 494 M]^+^, base peak: m/z = 357.

**3h**

3-Amino-12-chloro-1-(4-methoxyphenyl)-1*H*-benzo[*a*]pyrano[2,3-*c*]phenazine-2-carbo-nitrile (**3i,** new)

Yellow solid, M.P.: 271-274 °C; IR (KBr) ν = 3415, 3313, 3196, 2835, 2195, 1669, 1605, 1509, 1386, 1251, 1173, 1054, 1025, 925, 833, 768 and 651 cm^−1^; ^1^H NMR δ = 8.99 (d, *J* = 7.8 Hz, 1H), 8.33 (d, *J* = 8.0 Hz, 1H), 8.04 (d, *J* = 9.5 Hz, 1H), 7.85 (ddd, *J* = 24.3, 19.0, 10.8 Hz, 4H), 7.27 (d, *J* = 5.7 Hz, 4H), 6.75 (d, *J* = 8.0 Hz, 2H), 5.29 (s, 1H), 3.59 (s, 3H).; ^13^C NMR δ = 160.1, 158.3, 147, 144.6, 143.2, 140.3, 138.6, 136.3, 134.3, 131.6, 131.2, 129.9, 129.2, 127.3, 126.2, 125.3, 122.6, 120.7, 114.1, 58.6, 55.3 and 37; MS: m/z = 464 [M]^+^, base peak: m/z = 357.

**3i**

3-Amino-12-chloro-1-(*p*-tolyl)-1*H*-benzo[*a*]pyrano[2,3-*c*]phenazine-2-carbonitrile (**3j,** new)

Yellow solid, M.P.: 295-298 °C; IR (KBr) ν = 3456, 3321, 3179, 2882, 2190, 1665, 1603, 1594,1399, 1385, 1266, 1164, 1060 and 764 cm^-1^; ^1^H NMR δ = 9.06 (d, *J* = 7.9 Hz, 1H), 8.37 (d, *J* = 7.9 Hz, 1H), 8.20-7.78 (m, 5H), 7.38-7.14 (m, 4H), 7.00 (d, *J* = 7.6 Hz, 2H), 5.34 (s, 1H), 2.12 (s, 3H).; ^13^C NMR δ = 160.1, 146.7, 142.6, 141.3, 140.3, 136.2, 134.9, 131.2, 129.3, 128, 126.1, 125.3, 122.6, 120.7, 114.2, 58.6, 37.4 and 20.9; MS: m/z = 448 [M]^+^, base peak: m/z = 357.

**3j**

3-Amino-1-(3-nitrophenyl)-1*H*-benzo[*a*]pyrano[2,3-*c*]phenazine-2-carbonitrile (**3k**)

Yellow solid, M.P.: 281-284 °C; IR (KBr) ν = 3424, 3340, 3060, 2191, 1666, 1631, 1596, 1525, 1402, 1349, 1293, 1165 and 763 cm^-1^.

**3k**

3-Amino-1-(2-methoxyphenyl)-1*H*-benzo[*a*]pyrano[2,3-*c*]phenazine-2-carbonitrile (**3l**)

Yellow solid, M.P.: 272-276 °C; IR (KBr) ν: 3480, 3312, 3169, 3074, 2187, 1659, 1625, 1592, 1467, 1406, 1388, 1292, 1164, 1056, 1025, 848 and 757 cm^-1^.

**3l**

3-Amino-1-(2,4-dichlorophenyl)-1*H*-benzo[*a*]pyrano[2,3-*c*]phenazine-2-carbonitrile (**3m**)

Yellow solid, M.P.: 298-303 °C; IR (KBr) ν = 3480, 3312, 3169, 3074, 2187, 1659, 1625, 1592, 1467, 1406, 1388, 1292, 1164, 1056, 1025, 848 and 757 cm^-1^.

**3m**

3-Amino-1-phenyl-1*H*-benzo[*a*]pyrano[2,3-*c*]phenazine-2-carbonitrile (**3n**)

Yellow solid, M.P.: 300-303 °C; IR (KBr) ν = 3442, 3311, 3175, 3055, 2188, 1658, 1623, 1593, 1400, 1385, 1291, 1162, 1052, 760 and 703 cm^-1^.

**3n**

3-Amino-1-(4-chlorophenyl)-1*H*-benzo[*a*]pyrano[2,3-*c*]phenazine-2-carbonitrile (**3o**)

Yellow solid, M.P.: 295-298 °C; IR (KBr) ν = 3463, 3312, 3050, 2193, 1658, 1623,1592, 1472, 1386, 1292, 1163, 1053, 950, 761 and 751 cm^-1^.

**3o**

3-Amino-1-(3-bromophenyl)-1*H*-benzo[*a*]pyrano[2,3-*c*]phenazine-2-carbonitrile (**3p**)

Yellow solid, M.P.: 267-272 °C; IR (KBr) ν = 3444, 3317, 3178, 3055, 2199, 1660, 1626, 1593, 1475, 1401, 1290, 1161, 1054, 1018 and 787 cm^-1^.

**3p**

3‑Amino‑1‑(2‑chlorophenyl)‑1*H*‑benzo[*c*]pyrano[2,3‑*c*]phenazine‑2‑carbonitrile (**3q**)

Yellow solid, M.P.: 295-298 °C; IR (KBr) ν = 3469, 3439, 3312, 3050,2193, 1658, 1623,1592, 1472, 1386, 1292, 1163, 1053, 950, 761 and 751 cm^-1^; ^1^H NMR δ = 9.24 (d, *J* = 7.7 Hz, 1H), 8.46 (d, *J* = 7.9 Hz, 1H), 8.25 (d, *J* = 5.8 Hz, 1H), 8.02 (s, 1H), 7.86 (dd, *J* = 21.2, 5.3 Hz, 4H), 7.38 (d, *J* = 5.7 Hz, 3H), 7.24-7.18 (m, 2H), 7.10 (d, *J* = 4.3 Hz, 1H), 5.97 (s, 1H) ppm.

**3q**

3-Amino-1-(4-hydroxyphenyl)-1*H*-benzo[*a*]pyrano[2,3-*c*]phenazine-2-carbonitrile (**3r**)

Yellow solid, M.P.: 250-253 °C; IR (KBr) ν = 3469, 3439, 3312, 3050,2193, 1658, 1623,1592, 1472, 1404, 1386, 1292, 1163, 1053, 950, 761 and 751 cm^-1^.

**3r**

3-Amino-1-(3,4-dimethoxyphenyl)-1*H*-benzo[*a*]pyrano[2,3-*c*]phenazine-2-carbonitrile (**3s**)

Yellow solid, M.P.: 284-288 °C; IR (KBr) ν = 3414, 3333, 2964, 2931, 2839, 2193, 1664, 1593, 1515, 1462, 1264, 1166, 1139 and 754 cm^-1^.

**3s**

3-Amino-1-(4-nitrophenyl)-1*H*-benzo[*a*]pyrano[2,3-*c*]phenazine-2-carbonitrile (**3t**)

Yellow solid, M.P.: 283-285 °C; IR (KBr) ν = 3327, 3311, 3249, 3195, 2194, 1671, 1588, 1510, 1471, 1399, 1381, 1341, 1289, 1261, 1215, 1162, 1103, 1049, 1021, 823, 768 and 741 cm^-1^.

**3t**

3-Amino-1-(4-methoxyphenyl)-1*H*-benzo[*a*]pyrano[2,3-*c*]phenazine-2-carbonitrile (**3u**)

Yellow solid, M.P.: 267-271 °C; IR (KBr) ν = 3426, 3334, 3185, 2833, 2185, 1668, 1596, 1509, 1386, 1296, 1242, 1165, 1154, 1052 and 756 cm^-1^.

**3u**

3-Amino-1-(*p*-tolyl)-1*H*-benzo[*a*]pyrano[2,3-*c*]phenazine-2-carbonitrile (**3v**)

Yellow solid, M.P.: 291-294 °C; IR (KBr) ν = 3469, 3439, 3312, 3050, 2193, 1658, 1623,1592, 1472, 1386, 1292, 1163, 1053, 950, 761 and 751 cm^-1^.

**3v**

12-(4-Chlorophenyl)-3,3-dimethyl-2,3,4,12-tetrahydro-1*H*-benzo[*b*]xanthene-1,6,11-trione (**4a**)

Yellow solid, M.P.: 220-225 °C; IR (KBr) ν = 3034, 2,965, 1,662, 1,617, 1,591, 1,357, 1186, 935 and 717 cm^-1^; ^1^H NMR δ = 8.40-6.78 (m, 8H), 5.10 (s, 1H), 2.68 (d, *J* = 4.5 Hz, 2H), 2.44-2.12 (m, 2H), 1.13 (d, *J* = 4.1 Hz, 3H), 1.04 (s, 3H) ppm.

**4a**

12-(4-Isopropylphenyl)-3,3-dimethyl-2,3,4,12-tetrahydro-1*H*-benzo[*b*]xanthene-1,6,11-trione (**4b**)

Yellow solid, M.P.: 223-226 °C; IR (KBr) ν = 3034, 2,957, 1,662, 1,617, 1,591, 1,378, 1,193, 836 and 721 cm^-1^; ^1^H NMR δ = 8.17-7.05 (m, 8H), 5.14 (d, *J* = 10.7 Hz, 1H), 3.01-2.50 (m, 3H), 2.50-2.05 (m, 2H), 1.46-0.54 (m, 12H) ppm.

**4b**

3,3-Dimethyl-12-(3-nitrophenyl)-2,3,4,12-tetrahydro-1*H*-benzo[*b*]xanthene-1,6,11-trione (**4c**)

Yellow solid, M.P.: 233-236 °C; IR (KBr) ν = 3090, 2958, 2931, 2869, 1667, 1620, 1594, 1527, 1348, 1196, 1301, 1115, 1015, 727 and 714 cm^-1^.

**4c**

12-(2-Hydroxyphenyl)-3,3-dimethyl-2,3,4,12-tetrahydro-1*H*-benzo[*b*]xanthene-1,6,11-trione (**4d**)

Yellow solid, M.P.: 235-237 °C; IR (KBr) ν = 2965, 2940, 2872, 1660, 1626, 1590, 1514, 1362, 1228, 1206, 1190, 1175 and 718 cm^-1^.

**4d**

12-(2,4-Dichlorophenyl)-3,3-dimethyl-2,3,4,12-tetrahydro-1*H*-benzo[*b*]xanthene-1,6,11-trione (**4e**)

Yellow solid, M.P.: 260-264 °C; IR (KBr) ν = 3073, 2961, 2928, 2869, 2833, 1722, 1668, 1650, 1620, 1594, 1469, 1378, 1276, 1203, 1046 and 724 cm^−1^.

**4e**

3,3-Dimethyl-12-phenyl-2,3,4,12-tetrahydro-1*H*-benzo[*b*]xanthene-1,6,11-trione (**4f**)

Yellow solid, M.P.: 228-233 °C; IR (KBr) ν = 3063, 3027, 2957, 2872, 1738, 1663, 1617, 1603, 1376, 1359, 1193, 1164, 1138, 730, 714 and 699 cm^−1^.

**4f**

12-(2-Chlorophenyl)-3,3-dimethyl-2,3,4,12-tetrahydro-1*H*-benzo[*b*]xanthene-1,6,11-trione (**4g**)

Yellow solid, M.P.: 246-249 °C; IR (KBr) ν = 3067, 2958, 2931, 2869, 1697, 1667, 1619, 1592, 1470, 1372, 1267, 1196, 1050, 1037, 757 and 725 cm^-1^.

**4g**

3,3-Dimethyl-12-(4-nitrophenyl)-2,3,4,12-tetrahydro-1*H*-benzo[*b*]xanthene-1,6,11-trione (**4h**)

Yellow solid, M.P.: 240-246 °C; IR (KBr) ν = 3073, 3040, 2961, 2931, 2866, 1681, 1660, 1617, 1593, 1516, 1345, 1205, 1193, 1114, 1015 and 719 cm^-1^.

**4h**

12-(3-Hydroxyphenyl)-3,3-dimethyl-2,3,4,12-tetrahydro-1*H*-benzo[*b*]xanthene-1,6,11-trione (**4i**)

Yellow solid, M.P.: 200-204 °C; IR (KBr) ν = 2960, 2940, 2876, 1659, 1621, 1590, 1514, 1375, 1361, 1228, 1204, 1190, 1175 and 720 cm^-1^.

**4i**

12-(4-Methoxyphenyl)-3,3-dimethyl-2,3,4,12-tetrahydro-1*H*-benzo[*b*]xanthene-1,6,11-trione (**4j**)

Yellow solid, M.P.: 270-274 °C; IR (KBr) ν = 2958, 2938, 2836, 1734, 1665, 1601, 1511, 1463, 1360, 1259, 1196, 1112, 1030 and 837 cm^-1^.

**4j**

3,3-Dimethyl-12-(p-tolyl)-2,3,4,12-tetrahydro-1*H*-benzo[*b*]xanthene-1,6,11-trione (**4k**)

Yellow solid, M.P.: 173-177 °C; IR (KBr) ν = 2958, 2925, 2866,1664, 1619, 1592, 1357, 1203, 1187, 1114, 1015, 935, 720 and 537 cm^−1^.

**5k**

12-(4-Hydroxyphenyl)-3,3-dimethyl-2,3,4,12-tetrahydro-1*H*-benzo[*b*]xanthene-1,6,11-trione (**4l**)

Yellow solid, M.P.: 202-205 °C; IR (KBr) ν = 2961, 2939, 2874, 1660, 1620, 1590, 1514, 1376, 1361, 1228, 1205, 1190, 1175 and 722 cm^-1^.

**4l**

FT-IR spectrum of **3a**


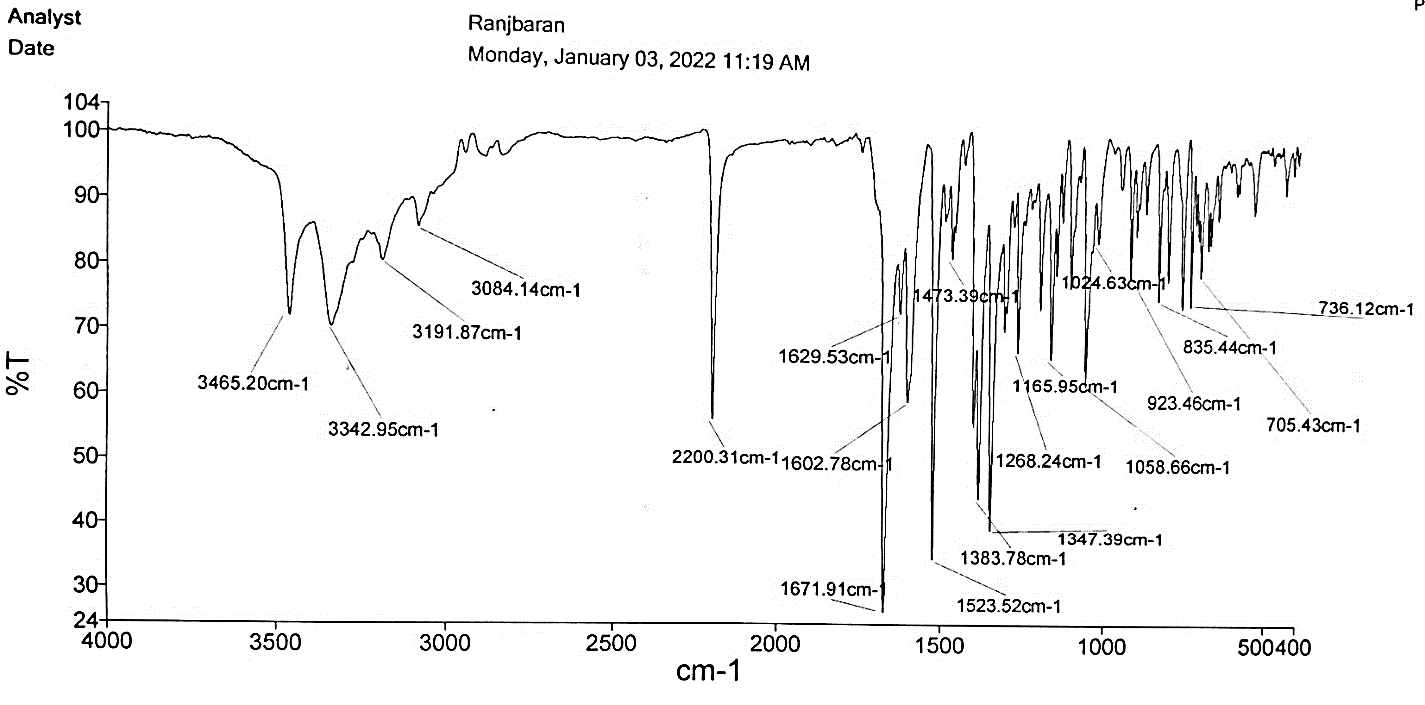


^1^H NMR spectrum of **3a**

^13^C NMR spectrum of **3a**

Mass spectrum of **3a**


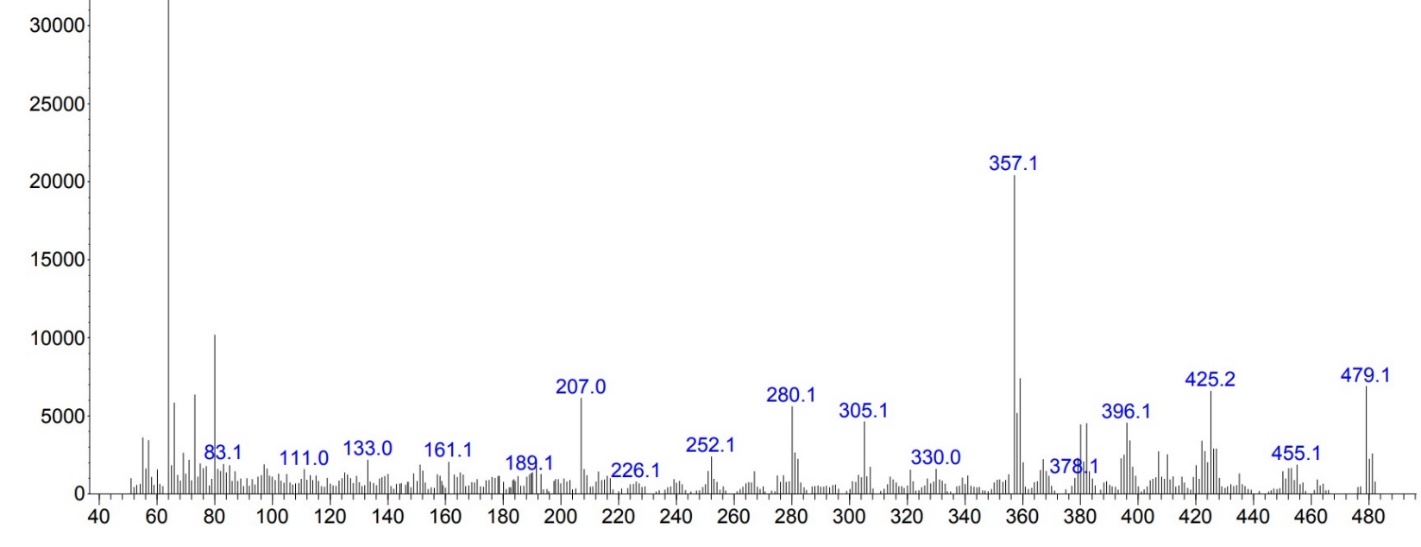


FT-IR spectrum of **3b**


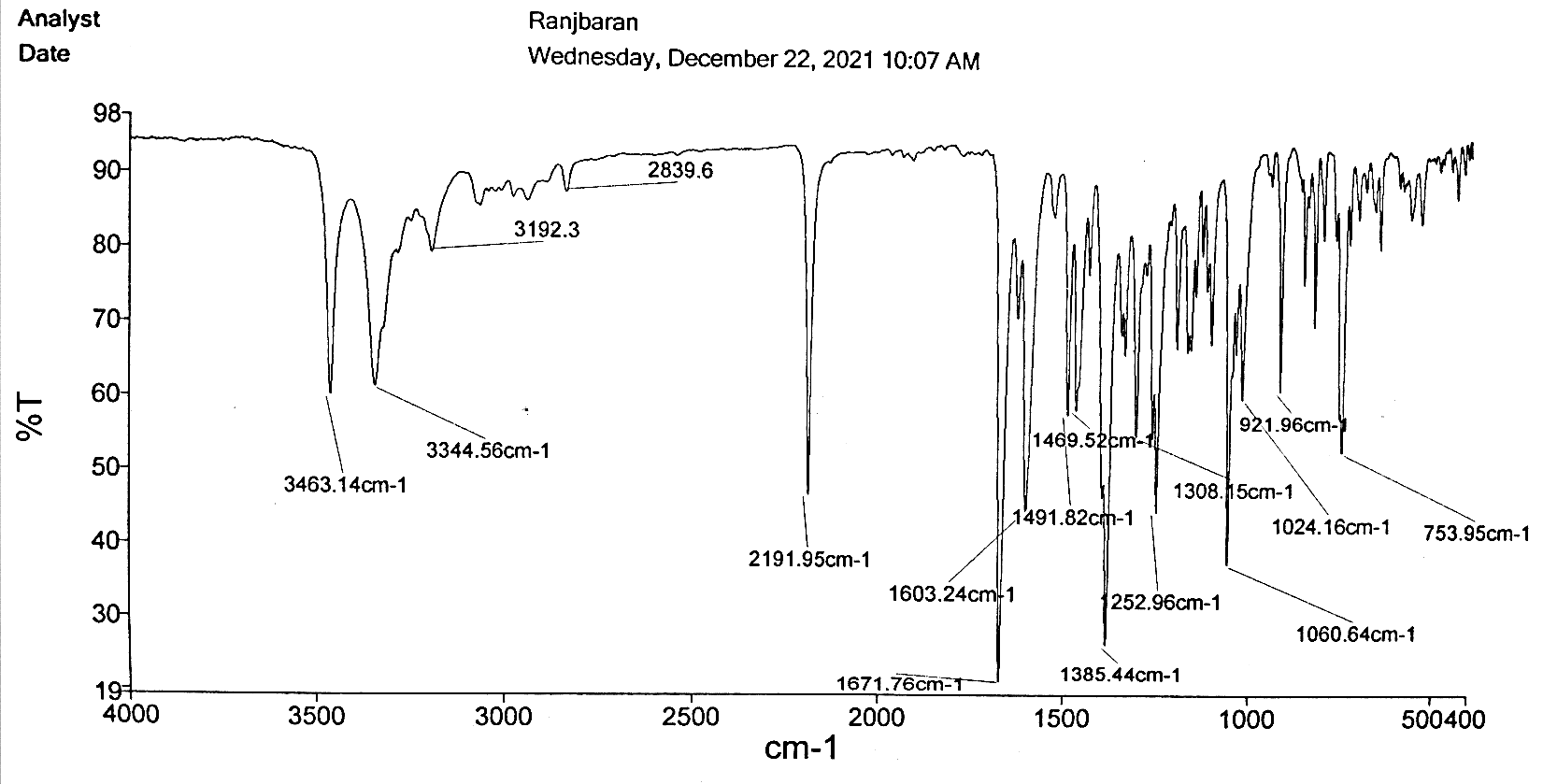


^1^H NMR spectrum of **3b**

^13^C NMR spectrum of **3b**

Mass spectrum of **3b**


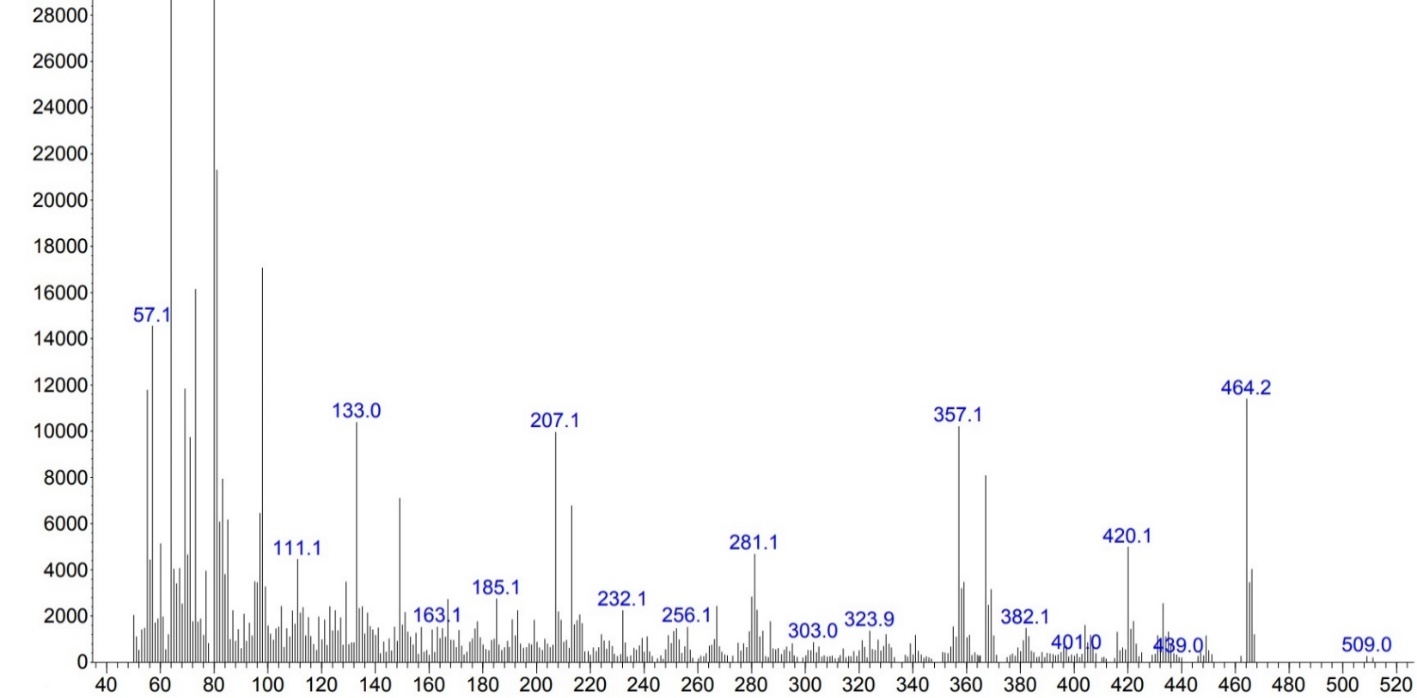


FT-IR spectrum of **3c**


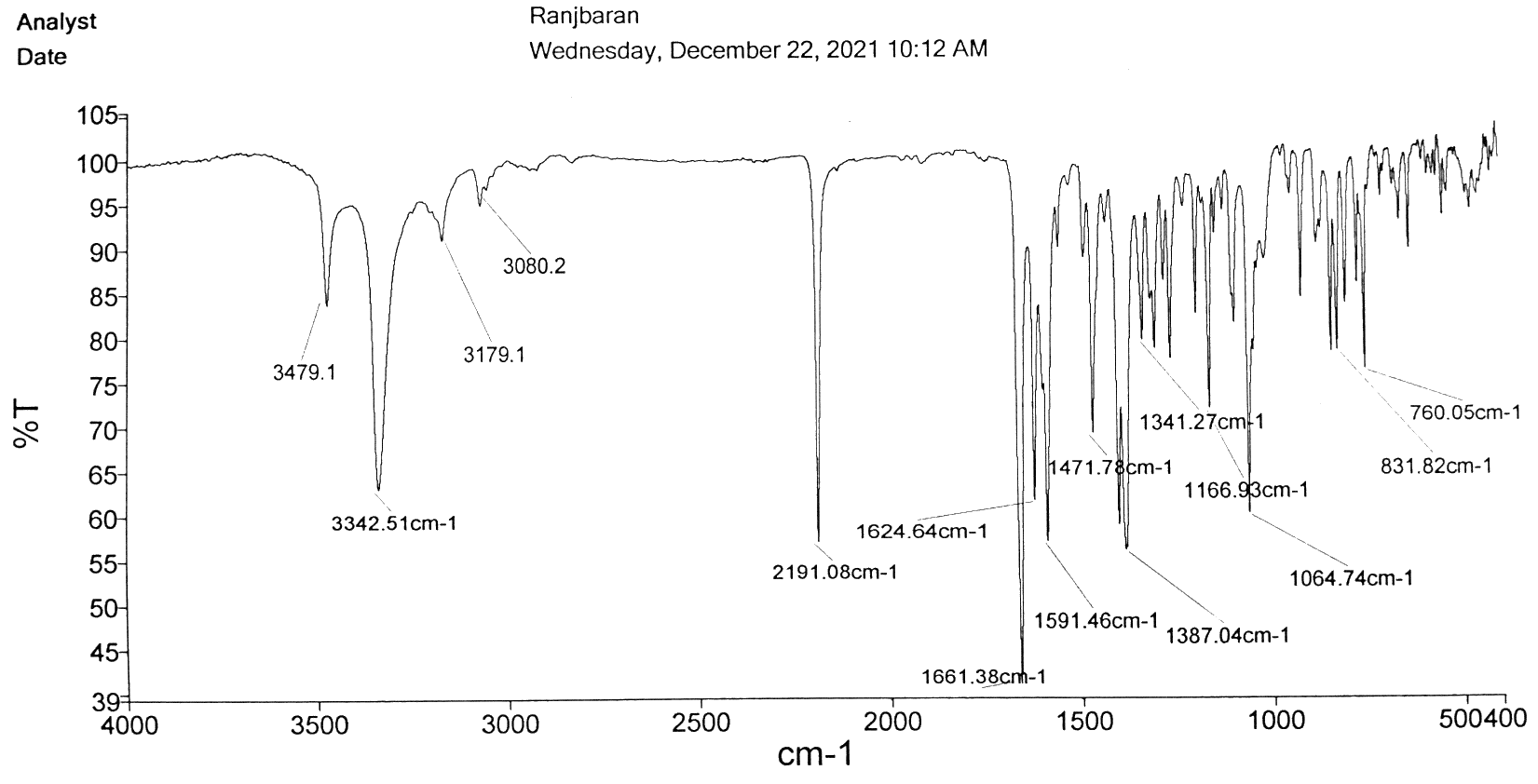


^1^H NMR spectrum of **3c**

^13^C NMR spectrum of **3c**

Mass spectrum of **3c**


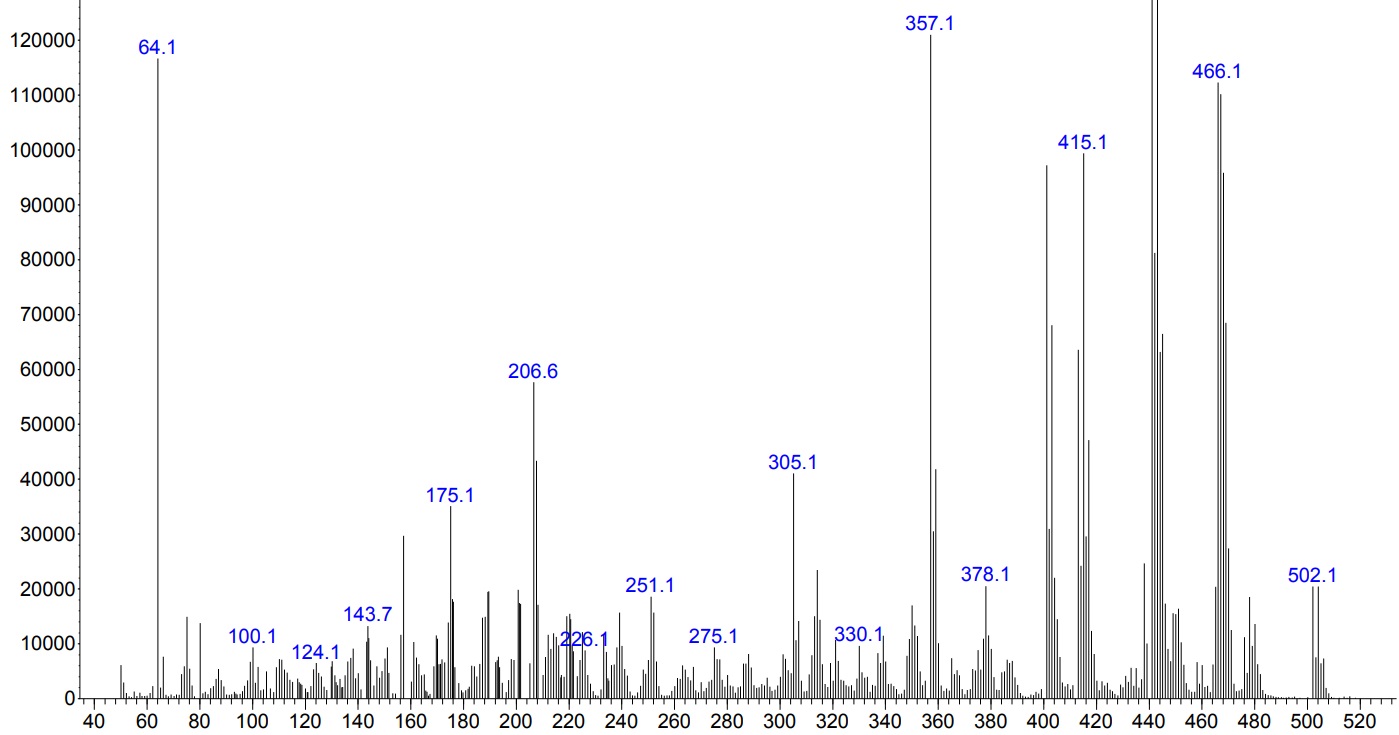


FT-IR spectrum of **3d**


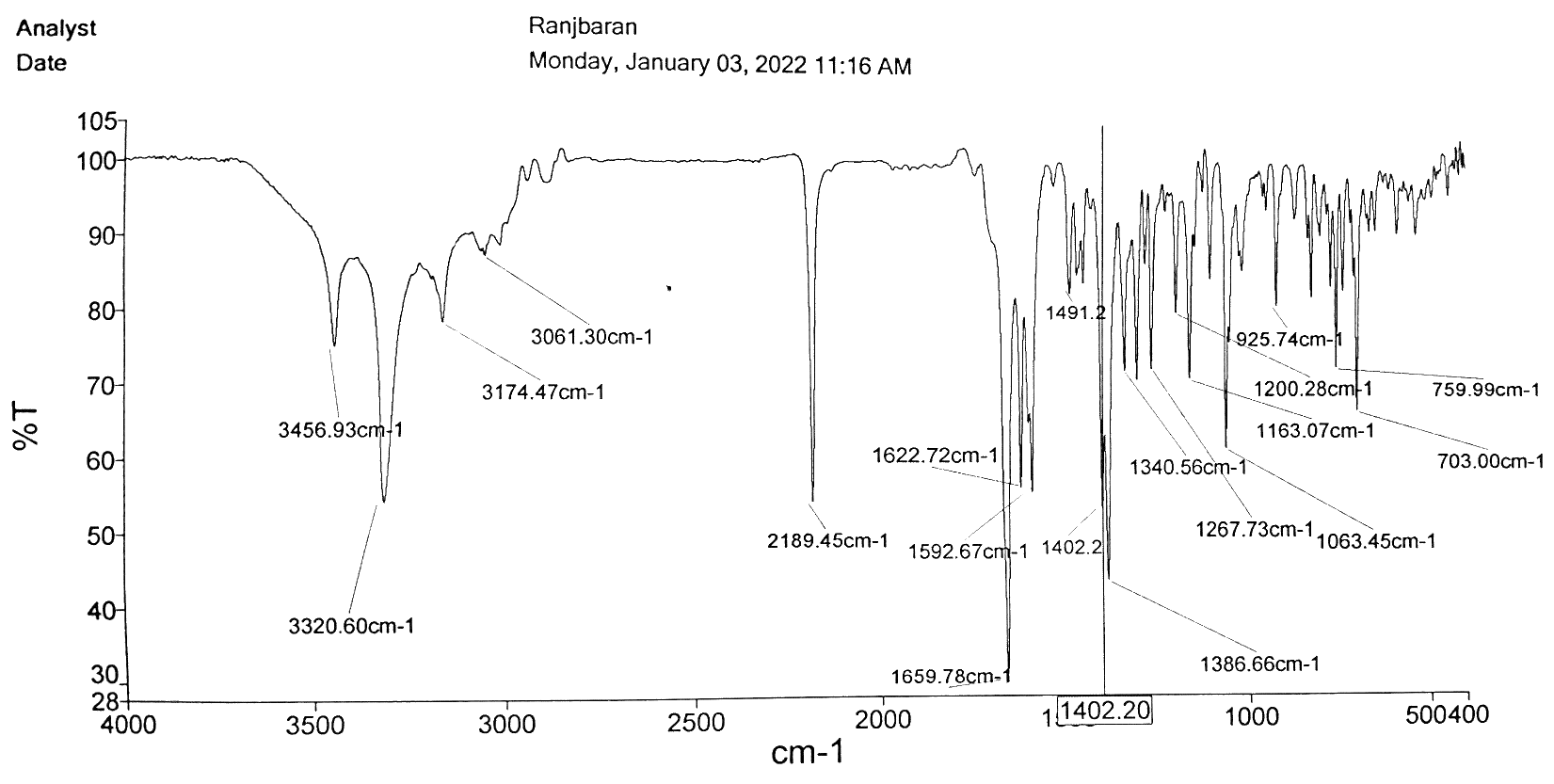


^1^H NMR spectrum of **3d**

^13^C NMR spectrum of **3d**

Mass spectrum of **3d**


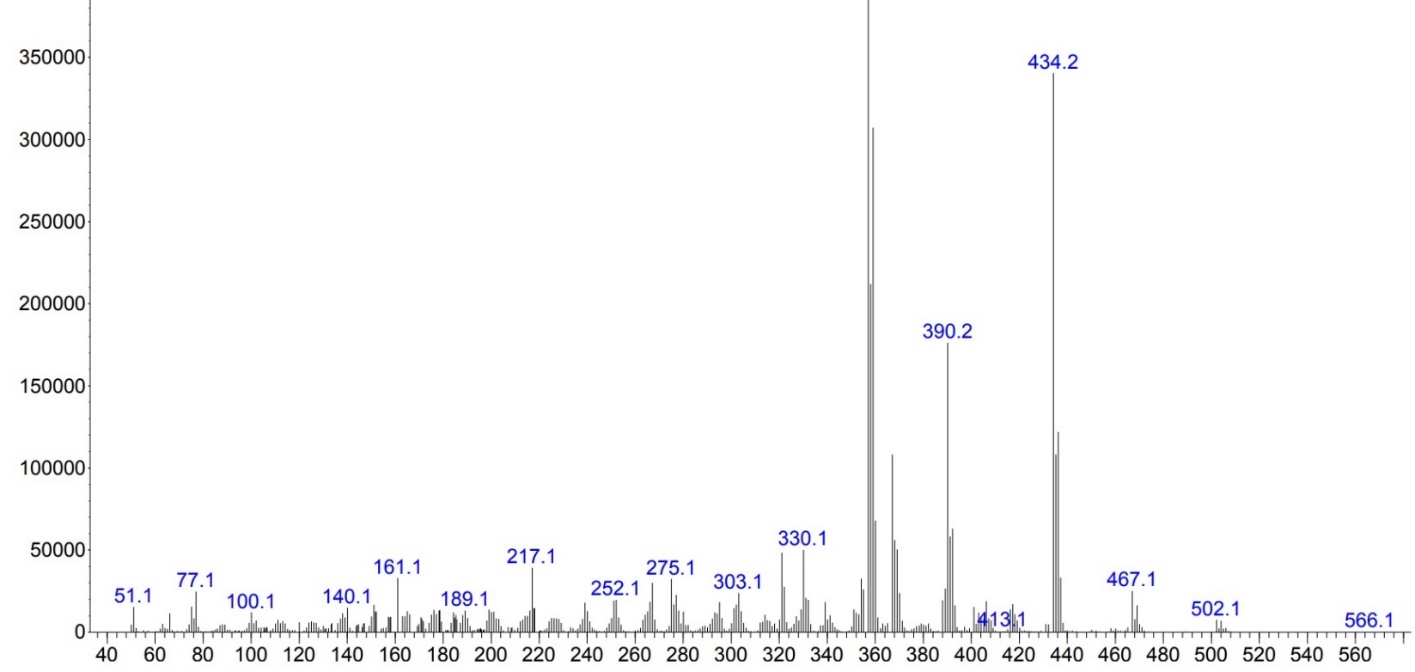


FT-IR spectrum of **3e**


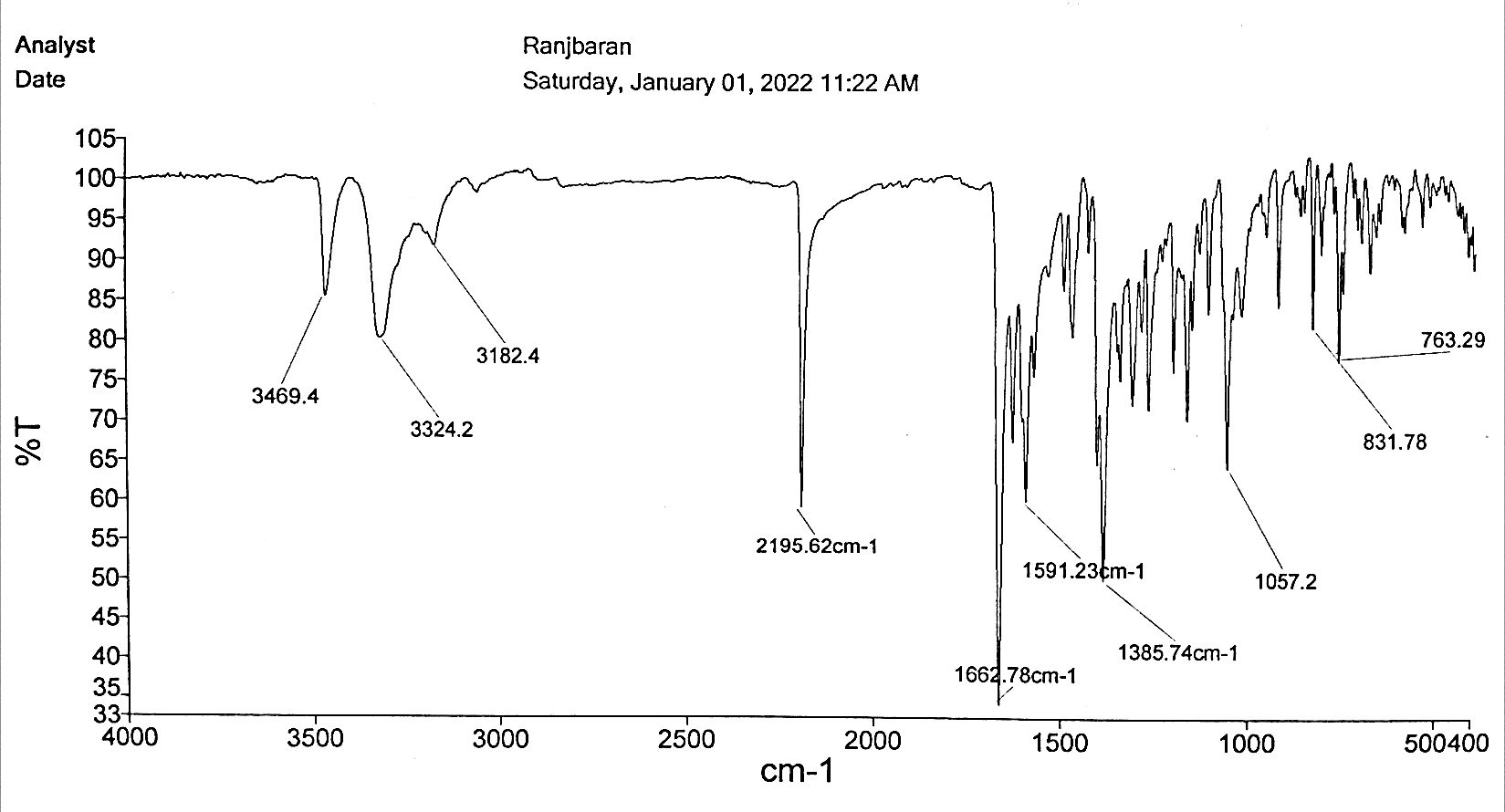


^1^H NMR spectrum of **3e**

^13^C NMR spectrum of **3e**

Mass spectrum of **3e**


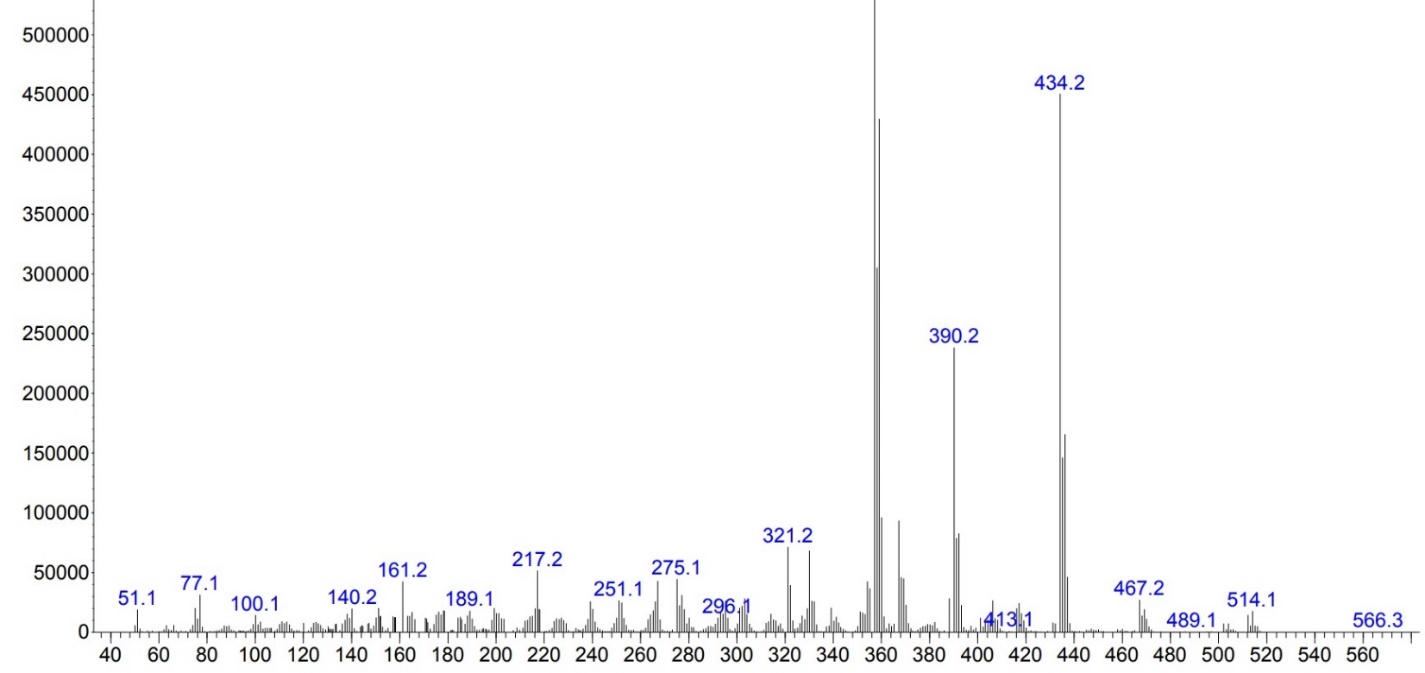


FT-IR spectrum of **3f**


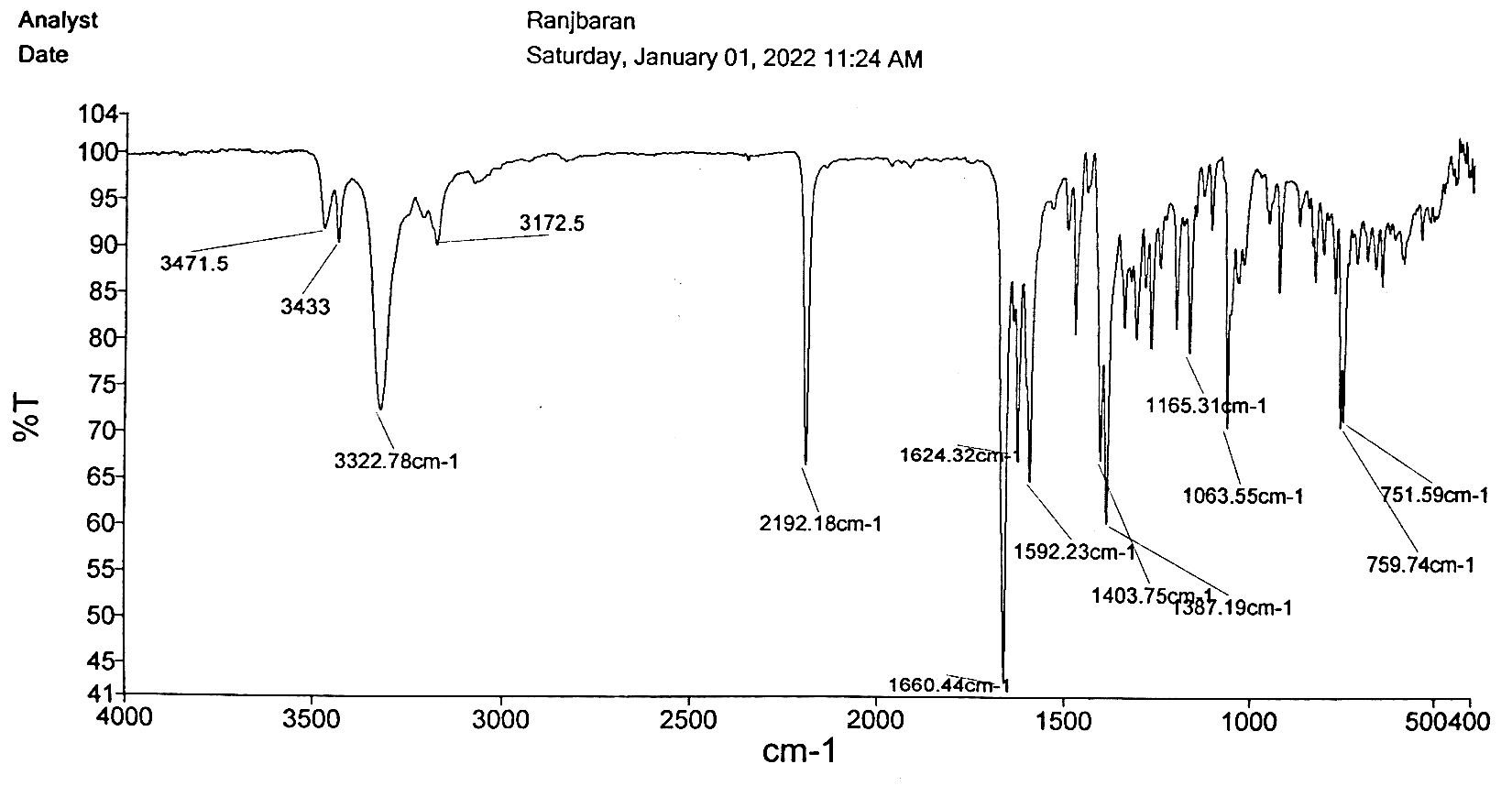


^1^H NMR spectrum of **3f**

^13^C NMR spectrum of **3f**

Mass spectrum of **3f**


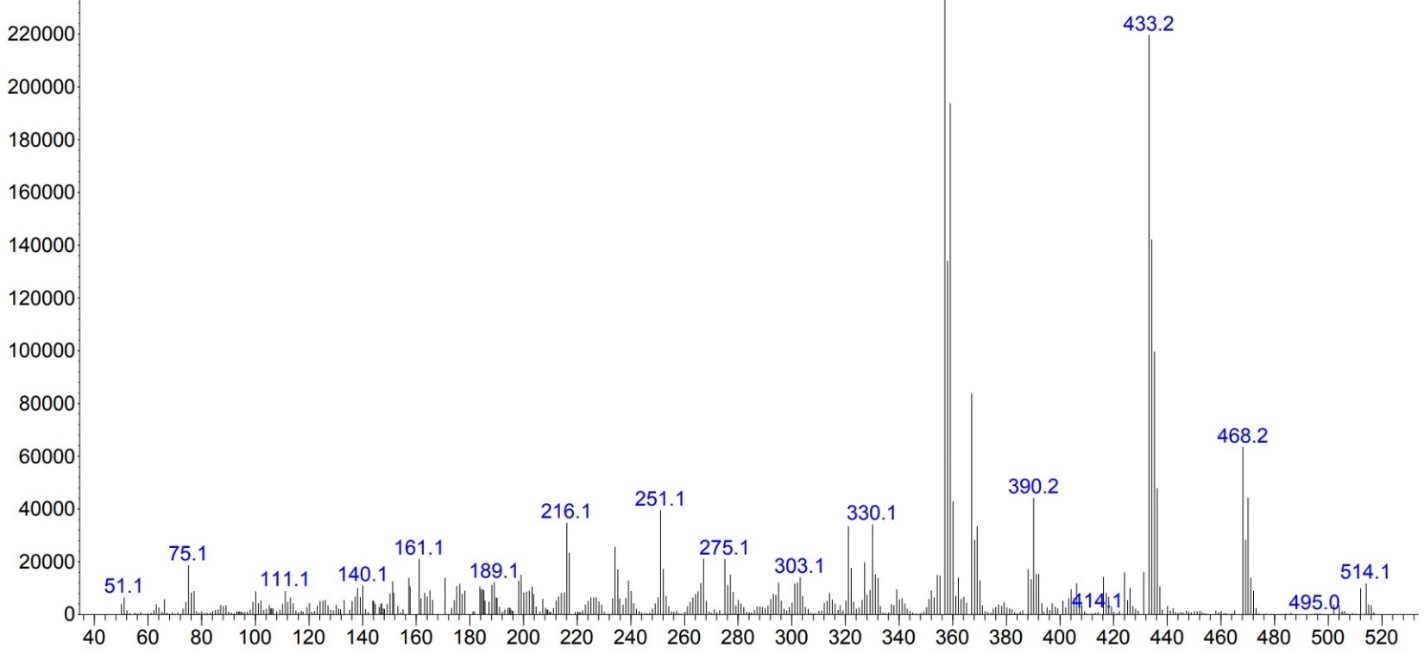


FT-IR spectrum of **3g**


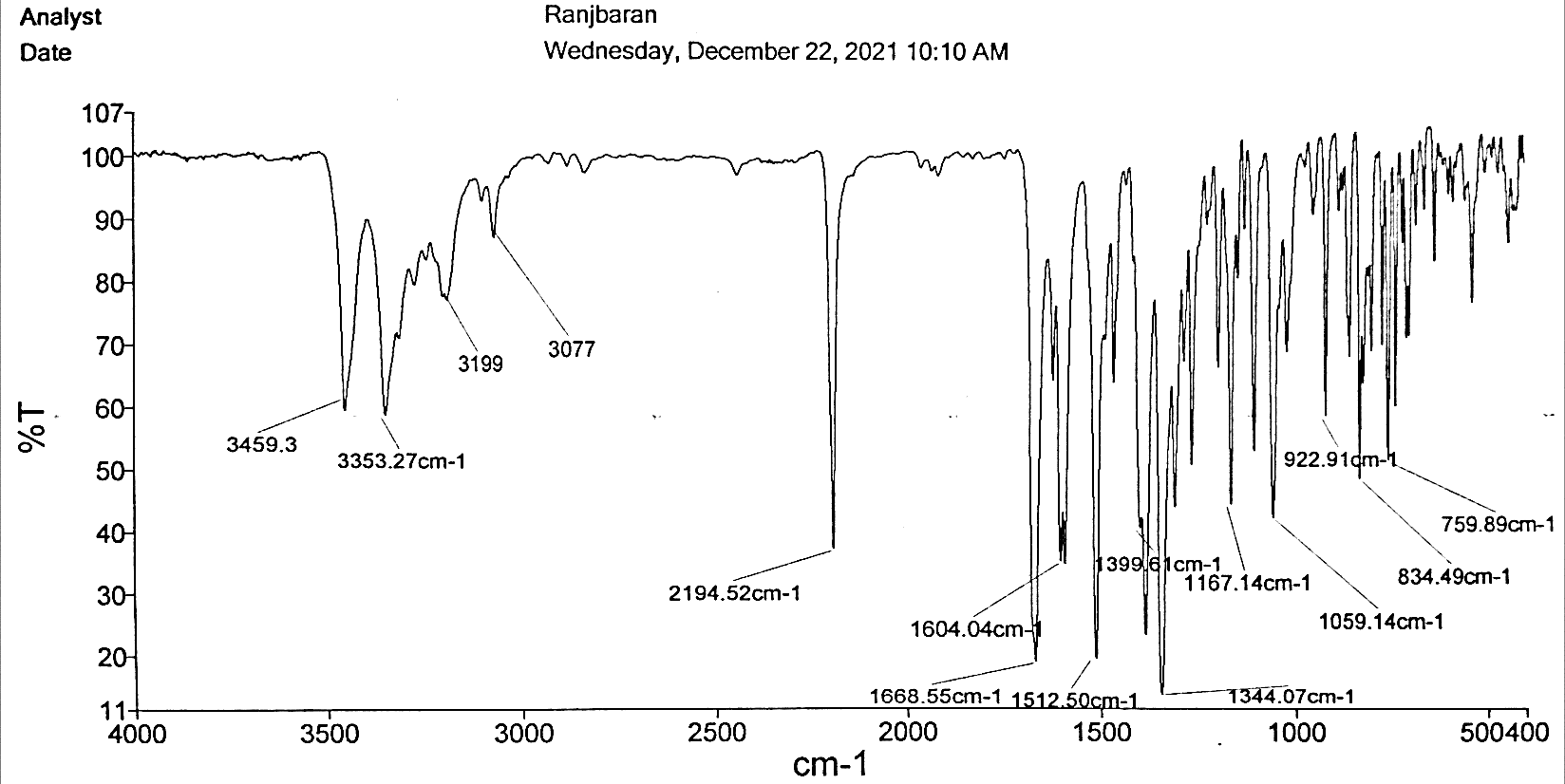


^1^H NMR spectrum of **3g**

^13^C NMR spectrum of **3g**

Mass spectrum of **3g**


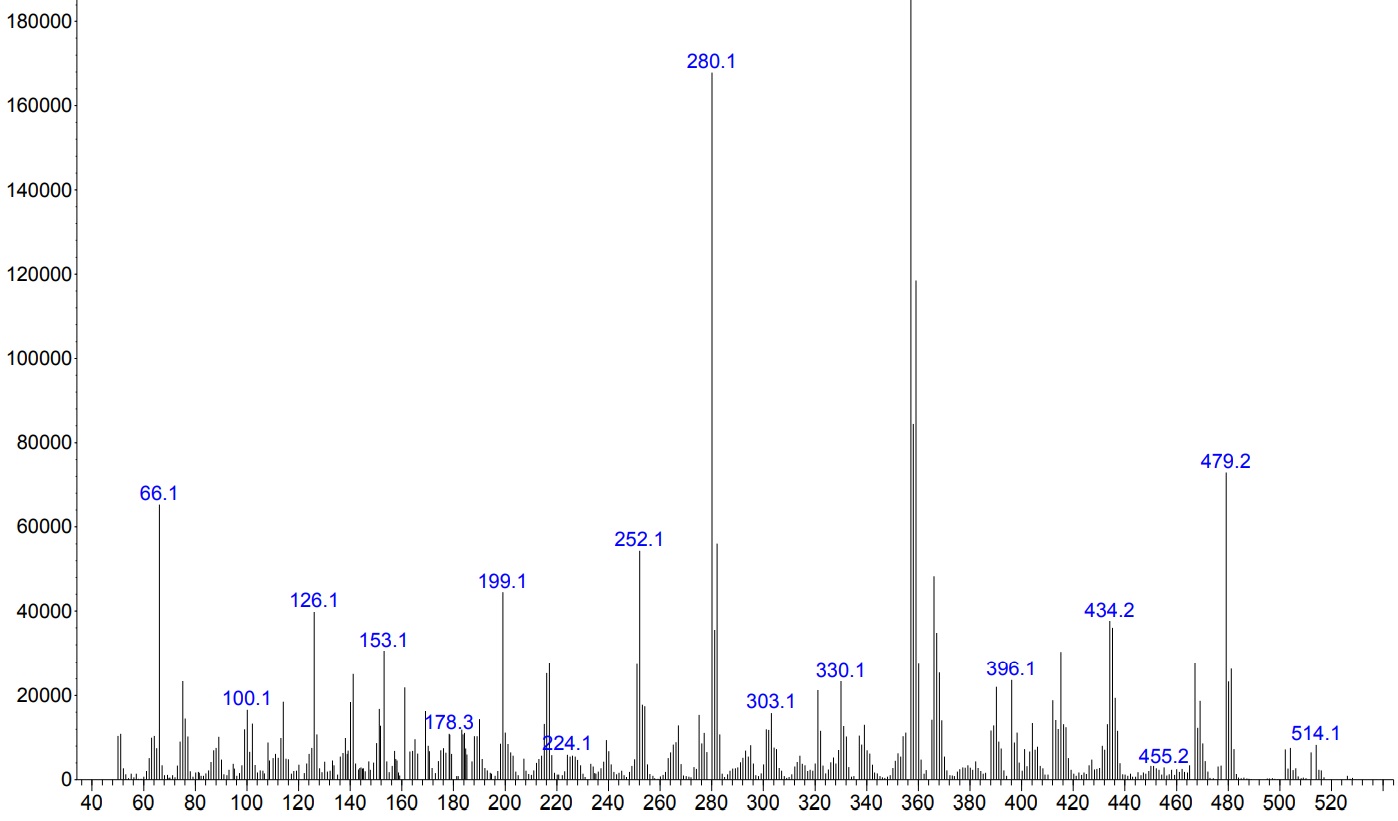


FT-IR spectrum of **3h**


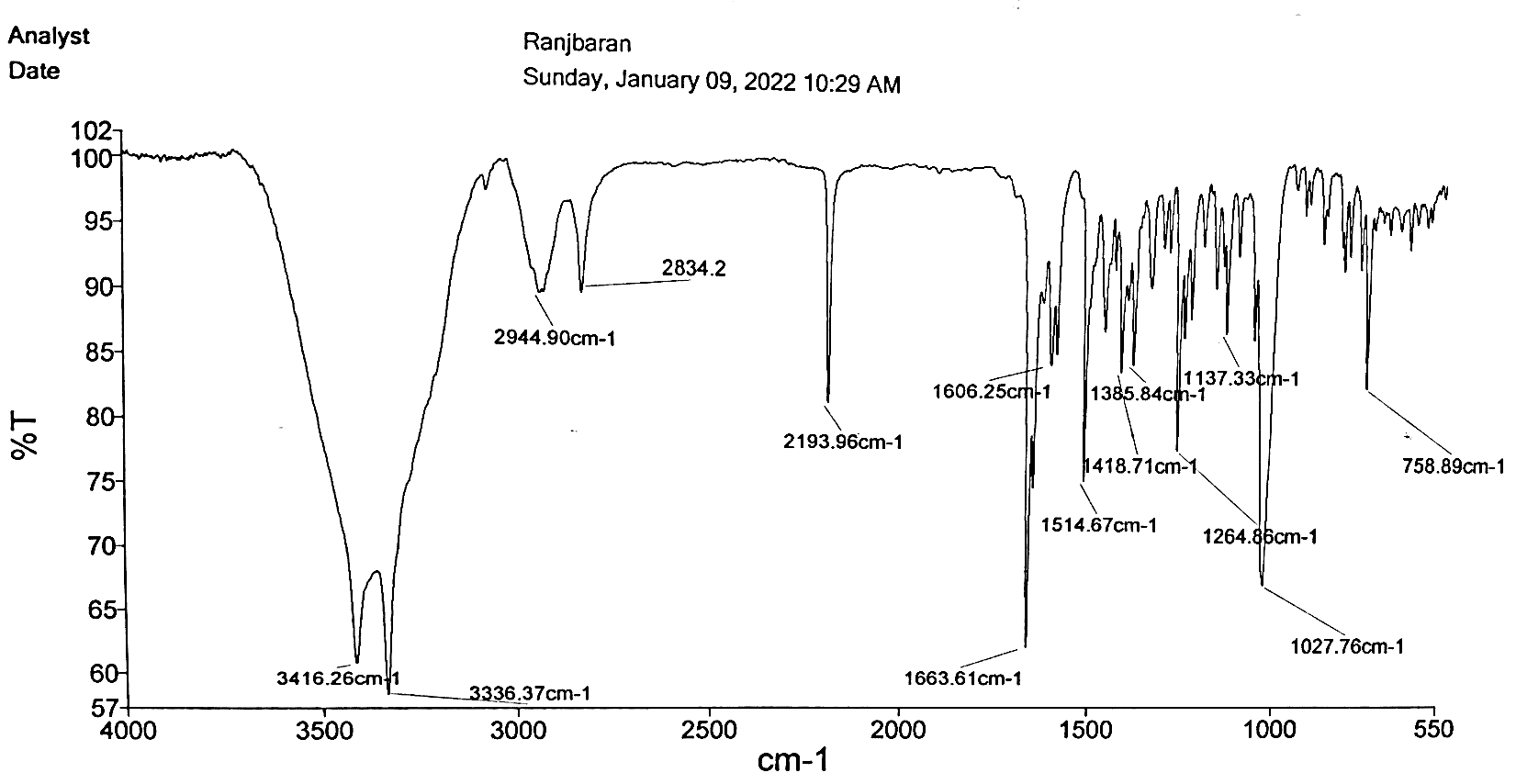


^1^H NMR spectrum of **3h**

^13^C NMR spectrum of **3h**

Mass spectrum of **3h**


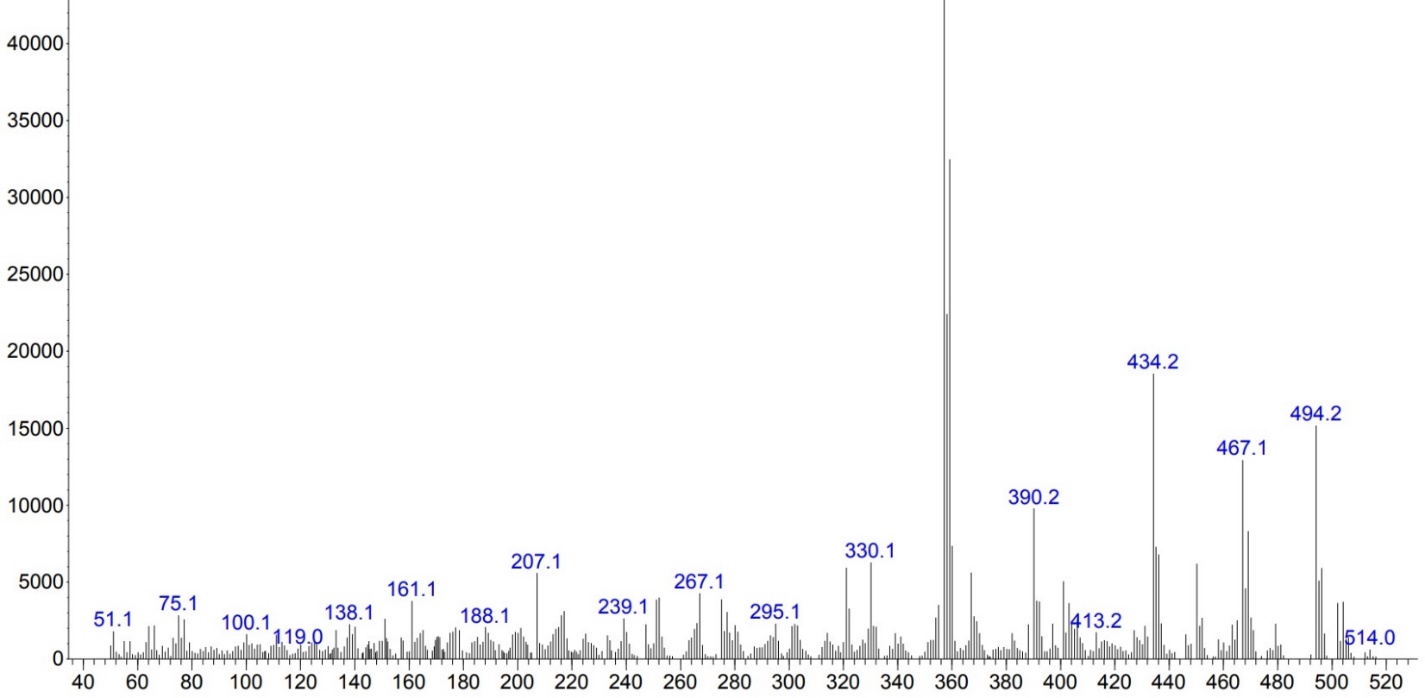


FT-IR spectrum of **3i**


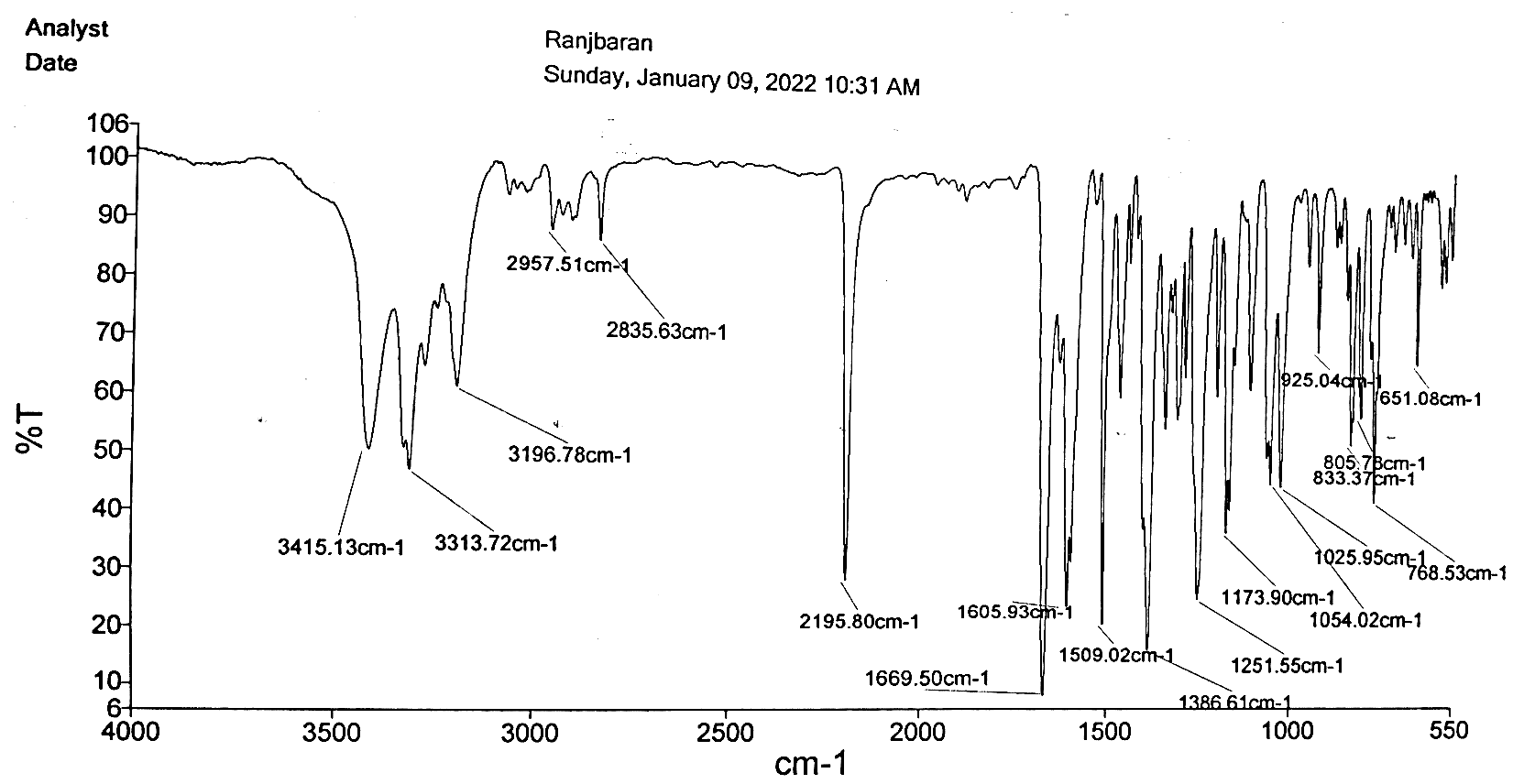


^1^H NMR spectrum of **3i**

^13^C NMR spectrum of **3i**

Mass spectrum of **3i**


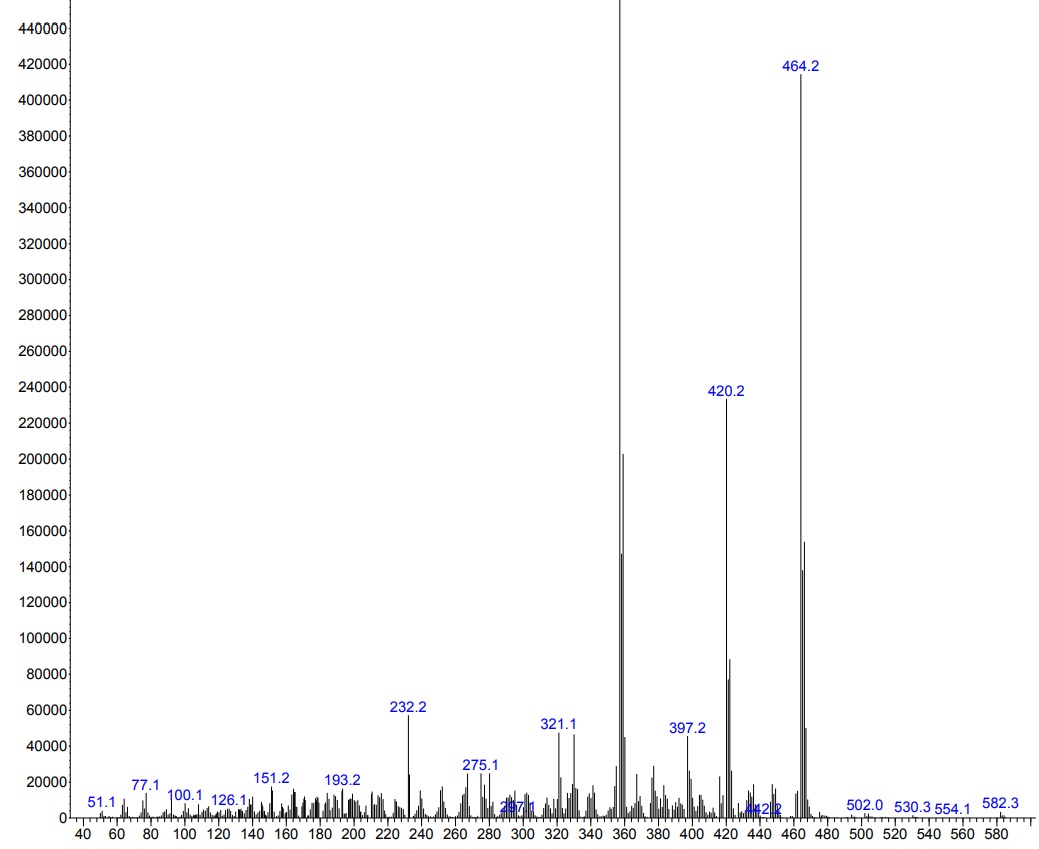


FT-IR spectrum of **3j**


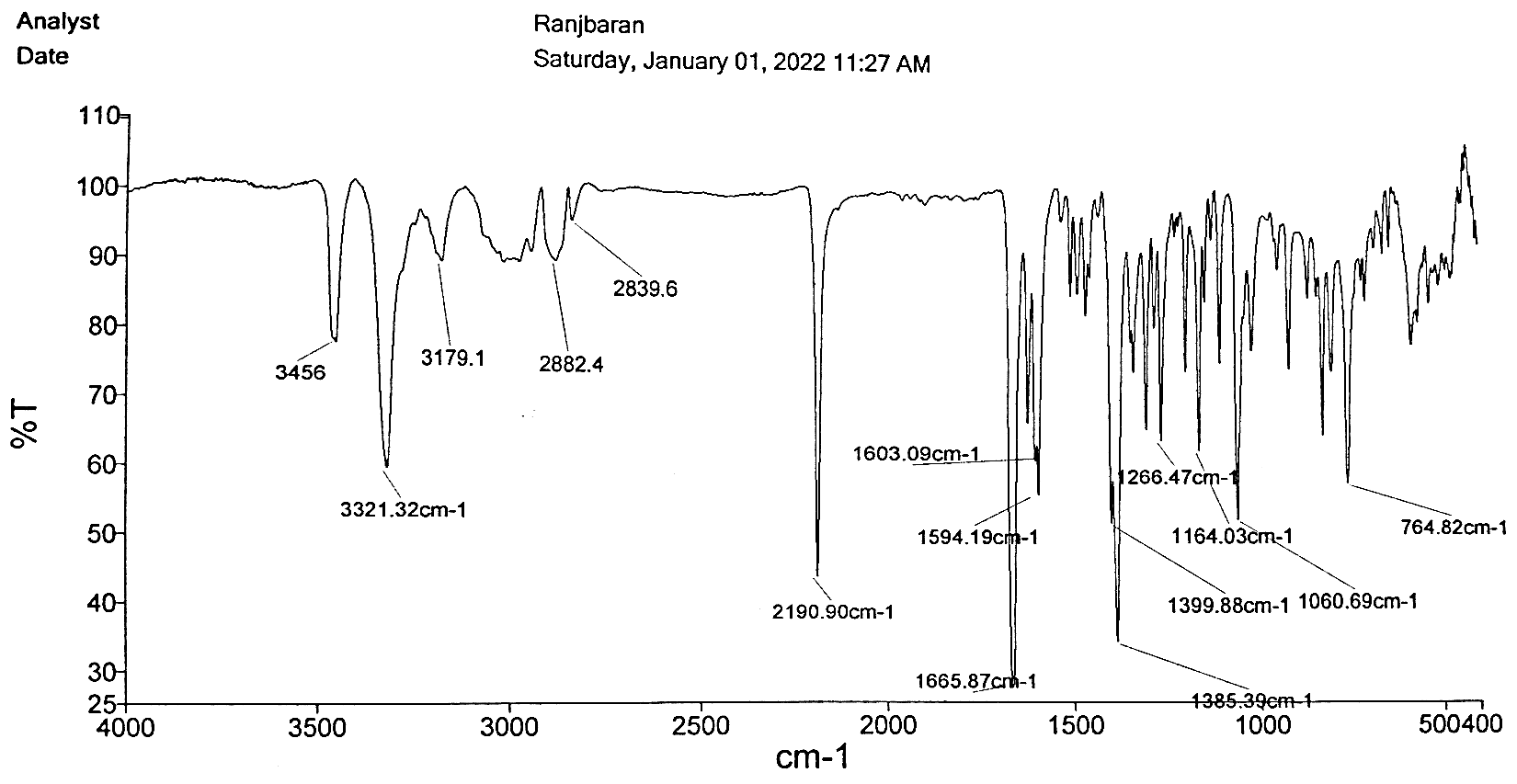


^1^H NMR spectrum of **3j**

^13^C NMR spectrum of **3j**

Mass spectrum of **3j**


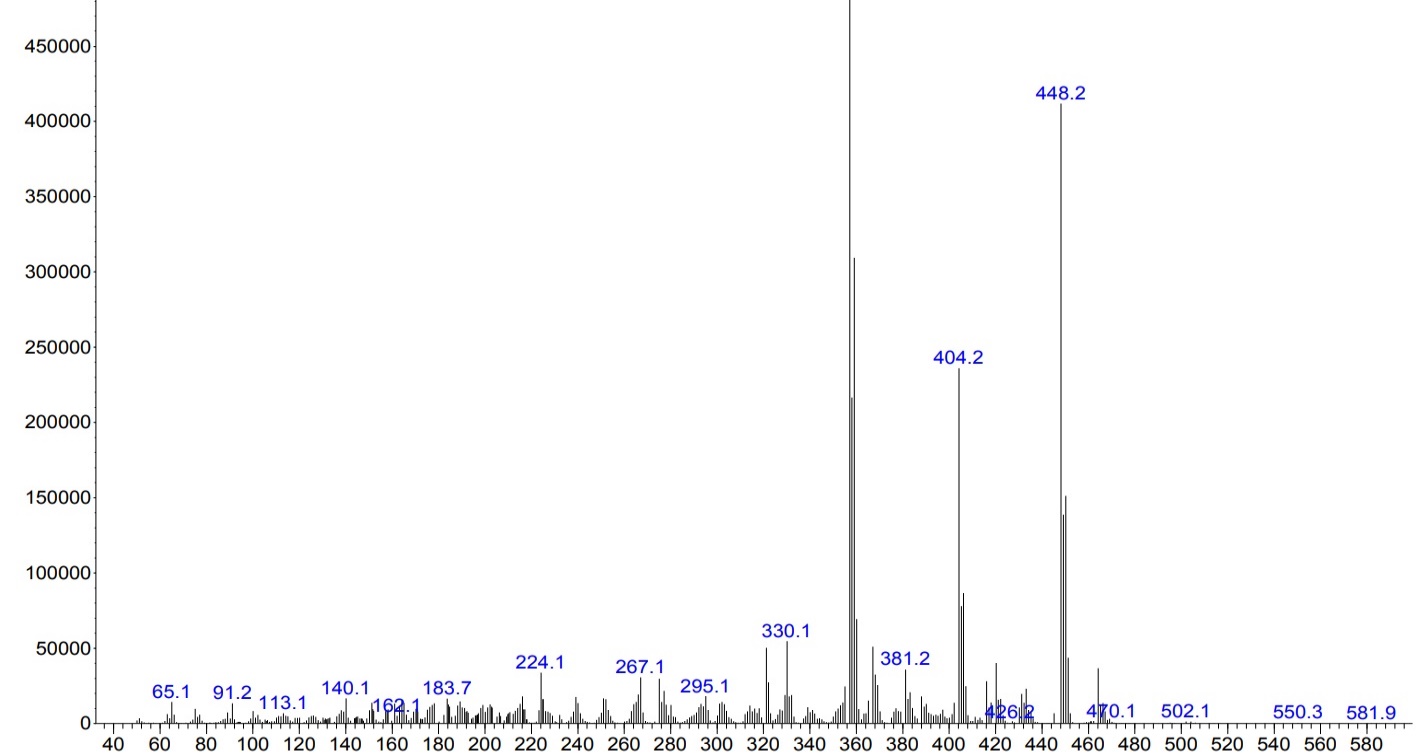


FT-IR spectrum of **3q**


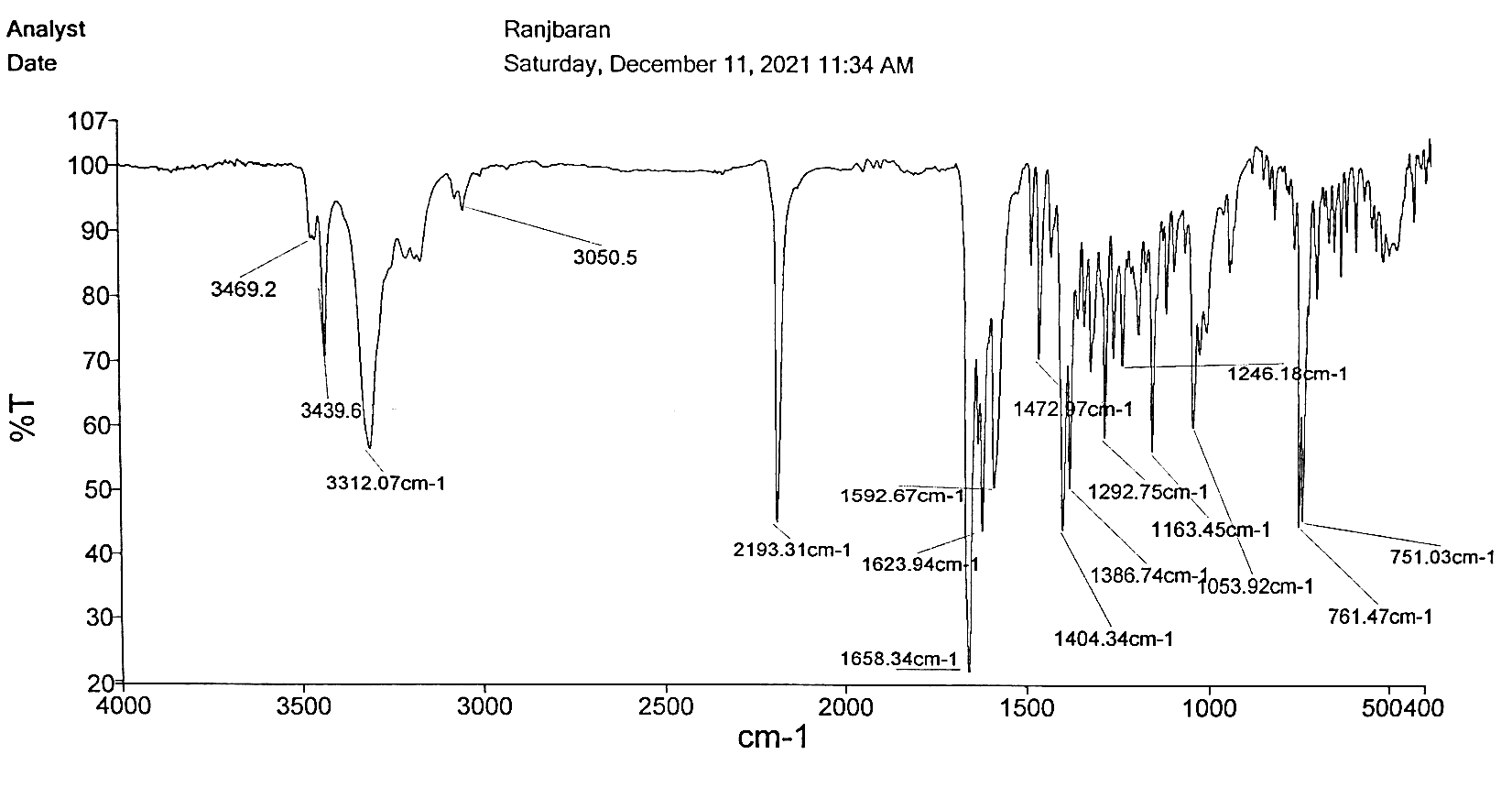


^1^H NMR spectrum of **3q**

FT-IR spectrum of **4a**


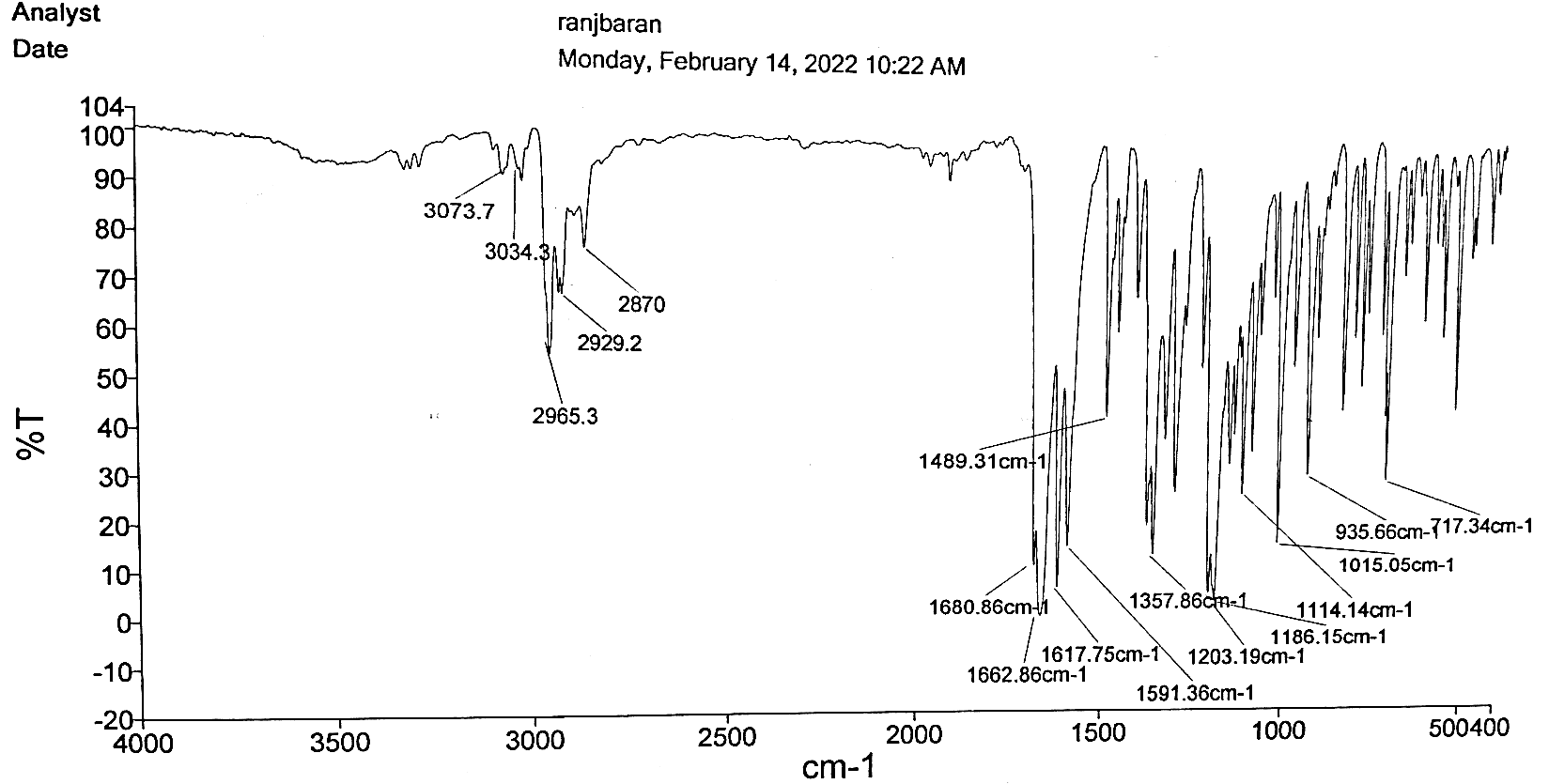


^1^H NMR spectrum of **4a**

FT-IR spectrum of **4b**


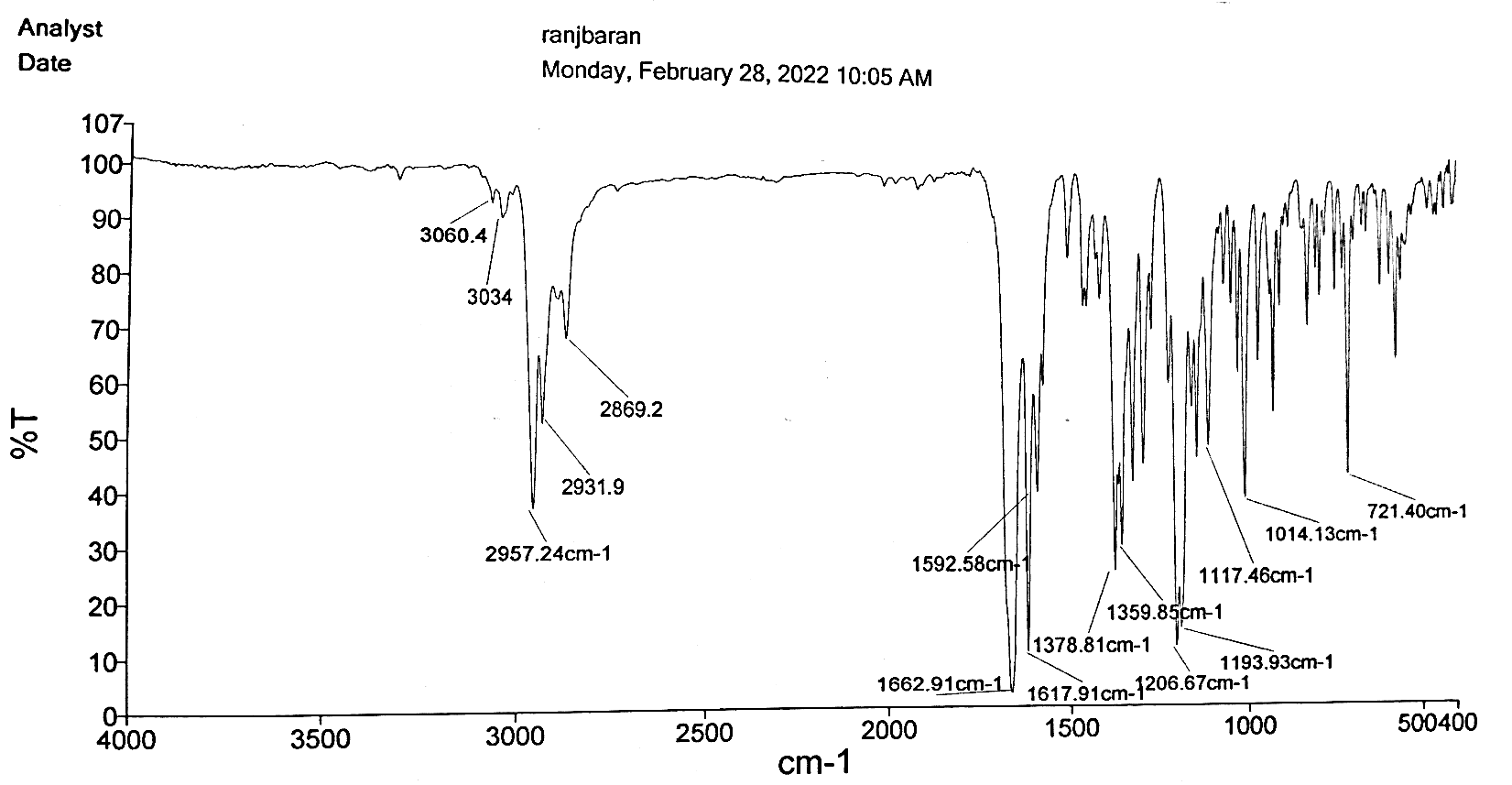


^\^

^\^

^1^H NMR spectrum of **4b**
